# Fecal microbiota composition, serum metabolomics, and markers of inflammation in dogs fed a raw meat-based diet compared to those on a kibble diet

**DOI:** 10.3389/fvets.2024.1328513

**Published:** 2024-04-17

**Authors:** Kris Hiney, Lara Sypniewski, Udaya DeSilva, Adel Pezeshki, Pratyaydipta Rudra, Parniyan Goodarzi, Erin Willis, Dianne McFarlane

**Affiliations:** ^1^Department of Animal and Food Sciences, Ferguson College of Agriculture, Oklahoma State University, Stillwater, OK, United States; ^2^Department of Clinical Sciences, College of Veterinary Medicine, Oklahoma State University, Stillwater, OK, United States; ^3^Department of Statistics, College of Arts and Sciences, Oklahoma State University, Stillwater, OK, United States; ^4^Department of Physiological Sciences, College of Veterinary Medicine, Oklahoma State University, Stillwater, OK, United States; ^5^Department of Large Animal Clinical Sciences, College of Veterinary Medicine, University of Florida, Gainesville, FL, United States

**Keywords:** canine, raw meat-based diet, raw diet, microbiome, metabolome, inflammatory markers

## Abstract

**Introduction:**

Despite the potential health risks associated with feeding raw and non-traditional diets, the use of these diets in dogs is increasing, yet the health outcomes associated with these diets is not well understood. This study investigates the effect of feeding dogs a kibble or raw meat-based diets on fecal microbiota composition, serum metabolomics and inflammatory markers.

**Methods:**

Clinically healthy dogs with a history of consuming either kibble (KD, *n* = 27) or raw meat-based diets (RMBD, *n* = 28) for more than 1 year were enrolled. Dogs were fed a standardized diet of either a single brand of KD or RMBD for 28 days. Serum and fecal samples were collected for analysis of microbiota, metabolomics, and inflammatory markers. Multiple regression analysis was performed for each of the metabolites and inflammatory markers, with feed group, age and BCS included as independent variables.

**Results:**

The fecal microbiota composition differed between the KD and RMBD groups. Beta-diversity and some indices of alpha-diversity (i.e., Shannon and Simpson) were different between the two diet groups. Sixty- three serum metabolites differed between KD and RMBD-fed dogs with the majority reflecting the differences in macronutrient composition of the two diets.

Fecal IAP, IgG and IgA were significantly higher in RMBD dogs compared to KD dogs, while systemic markers of inflammation, including serum c-reactive protein (CRP), galectin, secretory receptor of advanced glycation end-products (sRAGE), haptoglobin, and serum IgG were similar in dogs fed either diet.

**Discussion:**

Diet composition significantly affected fecal microbiota composition and metabolome. Although it had a potentially beneficial effect on local inflammatory markers, feeding RMBD had no impact on systemic inflammation. The influence of these changes on long term health outcomes provides an area for future study.

## Introduction

Many dog owners seek the healthiest diet to promote a long, quality life. The commercial dog food market is vast with formulated dry kibble dominating the market for its ease of use, meeting nutritional standards and shelf stability (1). Although generally considered healthy and safe, recent news reports of food recalls due to contamination with pathogenic microorganisms (bacteria, yeast, and fungi), mycotoxins, toxic commercial chemicals, and drugs such as melamine have heightened concerns among dog owners (1–7). Furthermore, grain-free, boutique dog foods, once believed by many consumers to be a more wholesome diet choice have been suggested to be correlated with development of dilated cardiomyopathy (8, 9), although the relationship between DCM and the feeding of grain-free diets is in dispute (10). For people, eating freshly prepared food is widely accepted as healthier and less inflammatory than a diet of heavily processed foods. Traditional pet foods are processed using heat treatments aimed at improving digestibility of included ingredients, extending shelf life and eliminating pathogens. However, high temperature processing may also alter bioavailability of some nutrients and increase the presence of byproducts such as advanced glycation end products (AGEs) (11–14). Furthermore, dry kibble production also requires the incorporation of additional ingredients such as binders or preservatives not found in fresh feeds which may impact health (15). Recently, feeding highly processed carbohydrate-based diets to juvenile dogs was identified as a risk factor for chronic enteropathies in later life (16).

In response, some pet owners are moving away from processed kibble dog food diets and implementing feeding fresh, dehydrated, freeze-dried and raw meat-based diets (17, 18). Reasons given for feeding fresh or raw diets include a nutrient profile that mimics ancestral diets, increased digestibility of nutrients, less chance of contaminants, and the belief it reduces chronic, inflammatory conditions such as allergies, avoids gastrointestinal disorders, improves dental health and reduces fecal output. (18, 19). However, raw or other unprocessed pet diets are not without risk to pets and pet owners. The increased presence of pathogenic bacteria in raw pet food diets has been clearly demonstrated, with case reports of serious food borne diseases in humans handling raw pet food as well as enteric diseases in pets consuming these product (20–29). The presence of antibiotic resistant bacterial populations in unprocessed pet foods has also been documented and may pose additional, critical risk to households handling such products (30). Although the risk of bacterial contamination is clear, the frequency of adverse health events in both pets and pet owners secondary to raw companion animal diets has been debated. In a recent survey of pet owners feeding a raw diet to their pet, the incidence of enteric diseases was suggested to be quite rare, although the study was limited as it relied on owner perception rather than confirmed diagnosis (31). Malnutrition may also occur in pets when owners fail to appropriately formulate home designed diets. Over 90% of dogs fed raw meat-based diets (RMBD) had at least one or more nutritional imbalances when examined by diet calculations (32). While case reports highlight the potential for this adverse outcome, there are no estimates of how common incidences of malnutrition may be in practice.

It is well known that diet is a strong driver of the gut microbiome, and the microbiome has a substantial role in overall health (33–36). Alterations in the microbiome may contribute to development of chronic disease (37, 38). Thus, the effects of raw feeding on the canine intestinal microbiome and determining whether it promotes beneficial or potentially harmful bacterial populations is of interest. Diet also impacts the metabolome, both directly through nutrients included or excluded in the diet and indirectly, through alterations in the microbiome. Metabolomic profiles can be used to screen for inflammatory disease in a non-targeted manner, while measurement of specific inflammatory biomarkers is a traditional approach to predictive chronic inflammation (39–42).

Considering the growing popularity and emerging marketing strategies to encourage the use of non-traditional diets despite the potential health risk to owners, it is worthwhile to evaluate the impact these diets have on canine health. To investigate the role of diet in intestinal and systemic health in dogs, the concentrations of fecal and serum inflammatory markers as well as metabolome and microbiota was compared in dogs fed a traditional kibble diet (KD) to those fed a raw meat-based diet (RMBD). We hypothesized that dogs fed RMBD would have alterations in their microbiota and metabolome that correlated with changes in fecal and systemic inflammatory markers.

## Materials and methods

This project was approved by Oklahoma State University’s Institutional Animal Care and Use Committee (ACUP# VM-18-26). Study recruitment was conducted through advertisement and social media campaigns targeting local and regional raw dog food cooperatives, dog events, canine performance groups, veterinary clinics and clients and employees of OSU’s Boren Veterinary Medical Hospital. Inclusion criteria required dogs to be systemically healthy adults (>1 year), > 9 kg, and to have been fed only a RMBD or an extruded commercial kibble diet (KD) for >1 year. Exclusion criteria included abnormal physical examination findings consistent with systemic disease, recent (within 4 months) administration of an antimicrobial, corticosteroid or immunomodulatory drug (e.g., Apoquel or Cytopoint), recent (within 2 weeks) vaccination, and pregnancy.

General health was confirmed via physical examination and baseline laboratory values by a single, blinded veterinary clinician. Free catch urine samples were collected from the dogs at the time of examination and urinalysis was completed on fresh urine samples by refractometry and urine test strip (Siemens Multistix 10 SG, Siemens Healthcare Diagnostics Inc., Tarrytown, NY, United States). Fecal samples were collected at the time of examination either through natural defecation or by manual rectal extraction by the veterinary clinician and evaluated by the Oklahoma Animal Disease and Diagnostic Laboratory for parasites.

All dogs were grouped according to their feeding method at enrollment (Table 1) based on owner provided diet history. As a large number of owners indicated they occasionally supplemented their dogs’ diets with treats, human food and leftovers, the diet was standardized before collecting samples. Dogs were placed on a 28-day restricted diet, limited to either a single brand of kibble (Purina Pro Plan Savor) or RMBD (Titan Blue, Ross Wells) and a single ingredient treat. KD dogs were fed a 50:50 blend of kibble of two protein sources (Shredded Beef and Rice: Shredded Chicken and Rice) to mimic the protein source of the RMBD more closely. All clients were supplied freeze-dried, single ingredient (liver) treats to be given if needed for training purposes. Diets were analyzed for dry matter, crude protein, fat, crude fiber, ash, minerals, and monosaccharides by Midwest Laboratories (Omaha, NE) (Supplementary Table S1). Amino acids (Supplementary Table S2) and fatty acid (Supplementary Table S3) profiles of diets were analyzed by Eurofins Scientific Inc. Nutrition Analysis Center (Eurofins, Des Moines, Iowa). Dog owners kept a weekly diet log on food consumption and to report any inadvertent food exposure. Owners were instructed to feed at a rate to maintain body weight and condition throughout the trial. Using the feed intake reported from weekly feeding logs completed by owners and nutritional analysis, daily dry matter mean intake was calculated for KD and RMBD fed groups for all nutrients (Supplementary Table S4), including individual amino acids (Supplementary Table S2) and fatty acids (Supplementary Table S3).

**Table 1:** Characteristics of enrolled dogs.

	KD	RMBD	*p*-value
Number enrolled	*n* = 27	*n* = 28	
Gender	*M* = 5; MC = 11; *F* = 0; FS = 11	*M* = 4; MC = 7; *F* = 6; FS = 11	*p* = 0.06
Age (mean ± SD)	4.5 ± 2.1 yrs	6.9 ± 2.6 yrs	*p* < 0.001
Weight (mean ± SD)	27.81 ± 13.6 kg	24.14 ± 11.1 kg	*p* = 0.28
BCS (mean ± SD)	5.1 ± 1.4	3.8 ± 1.2	*p* < 0.001
Breed (multiples)	BC = 3; Lab = 3; GD = 3; Aussie = 2; Mixed = 9	BC = 11; Rott = 4; ESS = 3; Lab = 2; Mixed = 5	
Breed (singles)	Corgi, Golden, Husky, Heeler, Greyhound, Staff Terr, Beagle	GSD, GSP, Mal, BM	
Primary diet brand at enrollment	ProPlan = 12; Hills science Diet = 10; Iams = 2; Taste of the Wild = 1; Diamond = 1; Pedigree = 1	Titan = 23; Texas Tripe = 4; Home preparation = 1	

Age, gender, body weight, and body condition score (BCS) were compared between the two feeding groups by Mann–Whitney test. BCS, body condition score; M, male; MC, male castrate; F, female; FS, female spayed; BC, Border Collie; Lab, Labrador Retriever; GD, Great Dane; Aussie, Australian Shepherd; Golden, Golden Retriever; Staff Terr, American Staffordshire Terrier; Rott, Rottweiler; ESS, English Springer Spaniel; GSD, German Shepherd Dog; GSP, German Shorthair Pointer; Mal, Malinois; BM, Bullmastiff.

Each dog returned on day 28 for physical examination and sample collection by the same blinded veterinary clinician. Clients were instructed to feed their dog half of the morning meal 120–240 min before their scheduled appointment. Blood samples were collected by jugular venipuncture into sterile vacuum red top blood tubes (BD Vacutainer^®^, Franklin Lakes, NJ, United States), placed on ice and transferred to the laboratory. The samples were centrifuged at 2000 x g for 10 min at 4°C, and the separated serum stored at −80°C. Fresh fecal samples were also collected (voided or digital extraction from rectum), snap frozen in liquid nitrogen, then stored at −80°C for later processing.

### Fecal microbiota

Fecal DNA isolation, amplicon sequencing, sequence data analysis, and taxonomic classification were done as previously described (43). PCR amplification, microbial amplicon sequencing and bioinformatics were performed at Novogene Corporation (Sacramento, CA, United States) using their standard data analysis pipeline in use as of October 2020 (detailed methods included in Supplementary Table S12). Following sequence data analysis, taxonomic classification was performed, and rarefaction curves were generated. Alpha diversity was assessed using Chao1, Observed-species, Shannon, and Simpson indices. All these indices were calculated with QIIME (Version 1.7.0) and displayed with R software (Version 2.15.3). Alpha diversity boxplots were formed to analyze difference of Alpha Diversity indices between groups. Two sample Wilcoxon tests were performed for analysis of significance of difference between groups. The beta diversity of bacterial populations was used to express differences between feeding groups in species complexity. Beta diversity on both weighted and unweighted unifrac were calculated by QIIME software (Version 1.7.0). Cluster analysis was preceded by principal component analysis (PCA), which was applied using the FactoMineR package and ggplot2 package in R software (Version 2.15.3). Principal Coordinate Analysis (PCoA) of weighted or unweighted unifrac was performed to get principal coordinates and visualize from complex, multidimensional data. PCoA analysis was displayed by WGCNA package, stat packages and ggplot2 package in R software (Version 2.15.3). Anosim and Adonis were performed by R software (Vegan package: anosim function, and adonis function). AMOVA was calculated by mothur using amova function. Further, linear discriminant analysis (LDA) with effect size measurements was used for quantitative analysis of gut microbiota composition within dietary groups. LDA analysis was conducted using LEfSe software.

### Serum metabolomics

Serum metabolomics analysis was performed following published procedures (43) at West Coast Metabolomics Center (University California, Davis, Davis, CA, United States). Following data acquisition and data processing, the quantified metabolites values were reported as peak height. Kolmogorov–Smirnov test was applied to assess normality, then mean ± SD or median (interquartile range) was calculated for each identified metabolite. Resulting *p-*values were adjusted for multiple testing using the Benjamini–Hochberg procedure to control False Discovery Rate (44). Principle component analysis (PCA), pathway impact analysis and hierarchical clustering analysis were performed using MetaboAnalyst 3.092 (available online at: http://www.metaboanalyst.ca/faces/ModuleView.xhtml).

### Inflammatory markers

Fecal samples were homogenized in protease inhibitor, spun at 12,000 g for 20 min and the supernatants collected for analysis. Intestinal alkaline phosphatase activity (IAP) was determined using a previously described chromogenic enzyme assay as modified (45). Diluted fecal supernatants were reacted with *p*-nitrophenyl phosphate for 5 min, then stopped using 2 N NaOH. Chromogenic IAP activity was measured in an automated plate reader at 405 nM, using a serial dilution of shrimp alkaline phosphatase to create a standard curve. To confirm that the alkaline phosphatase (AP) activity was only from IAP, samples were treated with 10 mM phenylalanine, an inhibitor of IAP but not non-tissue specific AP. Fecal IgA and IgG was measured by canine specific ELISA (ICL, Inc., Portland, OR, United States). Protein concentration of the supernatants was measured (Bio-Rad, Hercules, CA, United States) and all fecal results expressed as enzyme activity (mIU/ul)/protein (mg/ml) (40).

Serum inflammatory marker galectin (RayBiotech Inc., Peachtree Corners, GA, United States), and serum IgG (ICL, Inc., Portland, OR, United States) were measured using canine specific commercial ELISAs. Serum sRAGE was measured using canine specific antibodies (R&D systems, Minneapolis, MN) and previously published methods (42). The concentration of serum haptoglobin was measured enzymatically using a multispecies, colorimetric assay (Phase™, TriDelta Development Ltd., County Kildare, Ireland). Serum C-reactive protein (CRP) was measured at the Gastrointestinal Laboratory at Texas A&M College of Veterinary Medicine (College Station, TX, United States).

### Statistics

Age, gender, and body condition score (BCS) were compared between the two feeding groups by Mann Whitney test. As age and BCS differed between the two groups (Table 1), multiple regression analysis was performed for each of the metabolites and inflammatory markers, with feed group, age and BCS included as independent variables. Resulting *p-*values were adjusted for multiple testing using the Benjamini–Hochberg procedure to control False Discovery Rate (44).

## Results

### Animals

Sixty-one dogs with a history of being fed a RMBD or kibble diet for ≥1 year were recruited. Six were excluded due to abnormalities on physical examination which deemed the animal unhealthy to participate. Excluded dogs (5 KD, 1 RMBD) had significant cardiovascular disease (*N* = 2), generalized skin infection (*N* = 2) and severe dental infection/periodontal disease (*N* = 2). Table 1 lists the specific diets of dogs prior to enrollment. Dogs were grouped (RMBD = 28, KD = 27) based on their diet at time of enrollment. Table 1 describes the animal signalment and physical characteristics of the two feeding groups (46). RMBD fed dogs were older (Age: KD = 4.5 yr., RMBD = 6.9 yr.; *p* < 0.001) and leaner (BCS: KD = 5.1, RMBD = 3.8; *p* < 0.001) than KD dogs. There were no differences in gender (*p* = 0.06), breed (*p* = 0.08) or purebred versus mixed breed (*p* = 0.23) between the two groups.

### Diet

Owners were instructed to feed at rates to maintain body condition score and weight throughout the 28- day feeding period. Caloric intake was determined through dietary logs, and the manufacturers reported caloric densities (kcal/kg) were used to calculate mean caloric intake (Supplementary Table S4). Caloric density (ME kcal/kg DM basis) of RMBD was greater than in KD (5,280 and 4,296 kcal/kg, respectively), however, owner feeding rates resulted in lower caloric intake per day in RMBD than KD (37.9 and 46.3 kcal/kg bwt/day, respectively). RMBD was higher in crude protein on a DM basis (RMBD = 49.7%, KD = 30.8%) and fat (acid hydrolysis) (RMBD = 43.5%, KD = 18.8%), while the KD was higher in total starch (KD = 32.3%, RMBD = 0.4%). Daily intake of nutrients per kg/bwt (Supplementary Table S4) were similar in protein (KD = 3.3 g /kg bwt, RMBD =3.6 g /kg bwt) but differed in fat (KD = 2.0 g/kg bwt, RMBD =3.2 g/kg bwt,) and starch (KD = 3.4 g/kg bwt, RMBD = 0.03 g/kg bwt). Intake of individual amino acids and fatty acids is shown in Supplementary Tables S3, S4.

Dietary intake of specific nutrients varied between the two groups, with RMBD having a higher intake per kg bwt of 10 /17 measured amino acids (tryptophan, methionine, alanine, aspartic acid, glycine, histidine, isoleucine, lysine, threonine, and valine) and a similar (≤20% difference) or lower intake for 7/17 amino acids (Supplementary Table S2). Despite this, plasma amino acid concentration was only statistically higher in RMBD compared to KD dogs for the 3 branched chain amino acids, as well as lysine, while it was lower in 7/17 amino acids (tryptophan, glutamine, glutamic acid, aspartic acid, phenylalanine, cystine and tyrosine; Supplementary Table S2). Amino acid derivatives higher in RMBD included alpha amino adipic acid, 2 aminobutyric acid, 2-hydroxybutanoic acid, *trans*-4-hyroxy-l-proline, 2-ketoisocaproic acid and kynurneic acid. Lipid, lipid derivatives, fatty acid and fatty acid metabolites which were higher in RMBD (which consumed more fat on a kg/bwt basis) included only phosphoethanolamine, 3-hydroxybutyric acid, 4-hydroxyphenylacetic acid, phytanic acid, linoleic acid, arachidonic acid, and 2-hydroxyhexanoic acid. Linoleic acid intake and arachidonic acid intake were substantially higher in RMBD dogs, with a twofold greater intake in linoleic acid and a fivefold greater intake in arachidonic acid (Supplementary Table S3).

### Fecal microbiota

**Figure 1:**
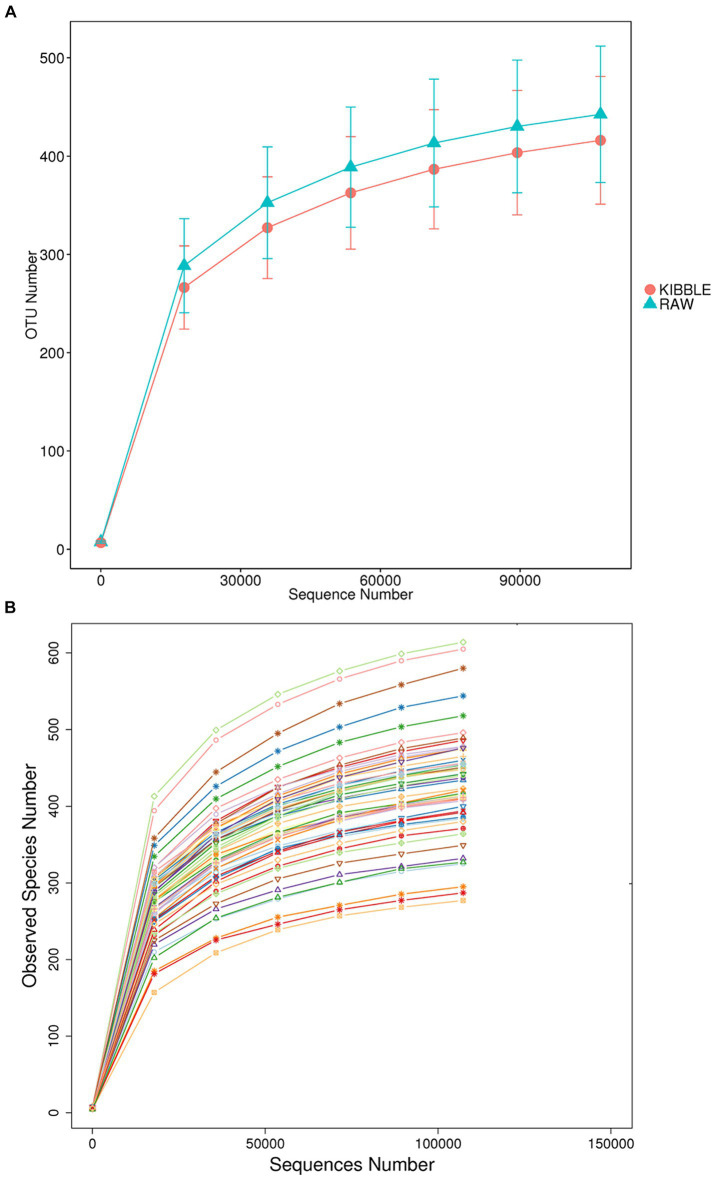
Fecal rarefaction curve analysis of dogs fed with kibble (KD) versus raw meat-based diets (RMBD). The rarefaction curves show the number of operational taxonomic unit (OTU) found as function of numbers of reads sampled when data were analyzed based on **(A)** dietary groups and **(B)** individual animals (each line represents an individual dog). *n* = 27 for KD and *n* = 28 for RMBD.

**Figure 2:**
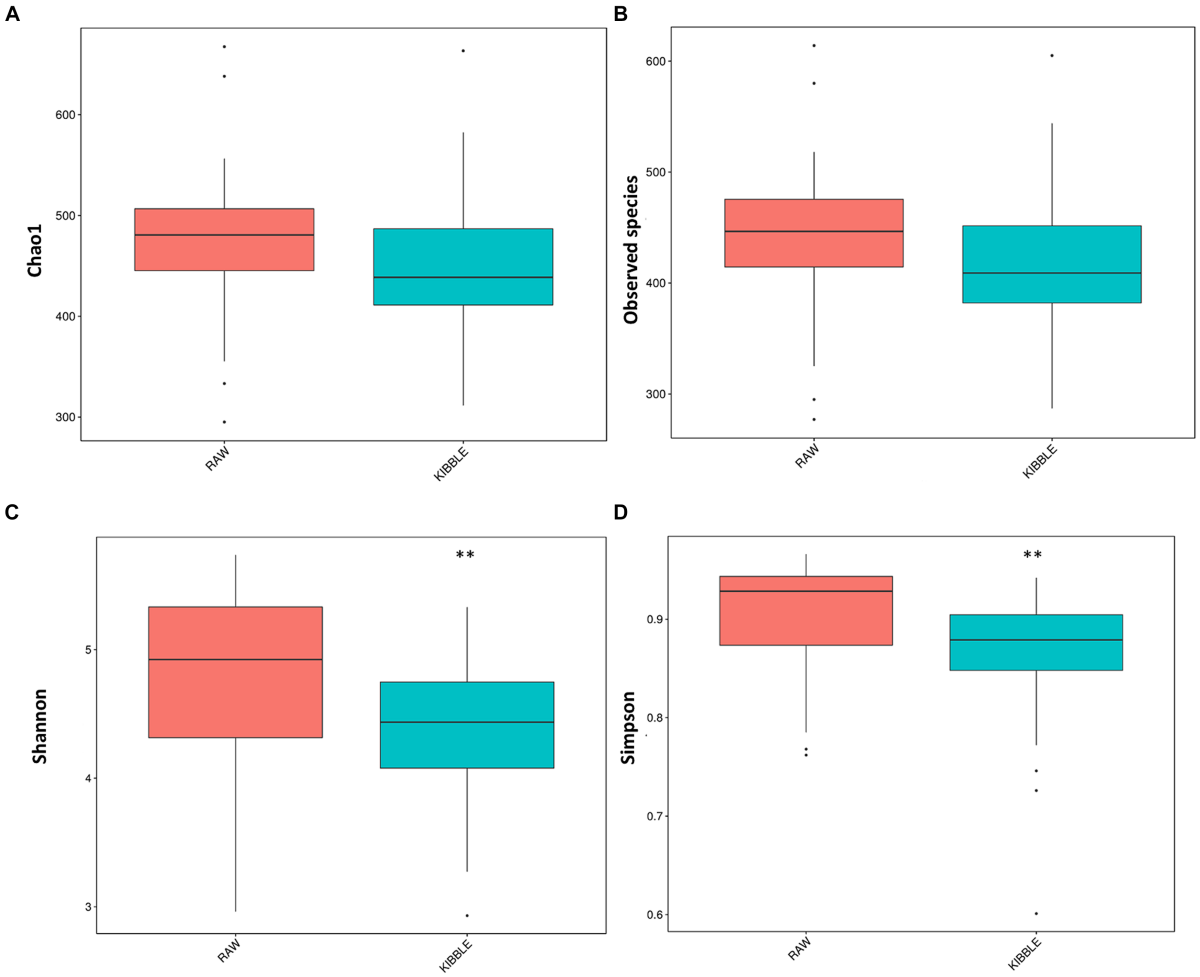
Alpha diversity indices for fecal bacterial community in dogs fed with kibble (KD) versus raw meat-based diets (RMBD). **(A)** Chao1, **(B)** Observed Species, **(C)** Shannon, and **(D)** Simpson. Median is shown with the line inside the box and outlier are shown as dots. ^**^Differences were considered significant at *p* ≤ 0.01. *n* = 27 for KD and *n* = 28 for RMBD.

**Figure 3:**
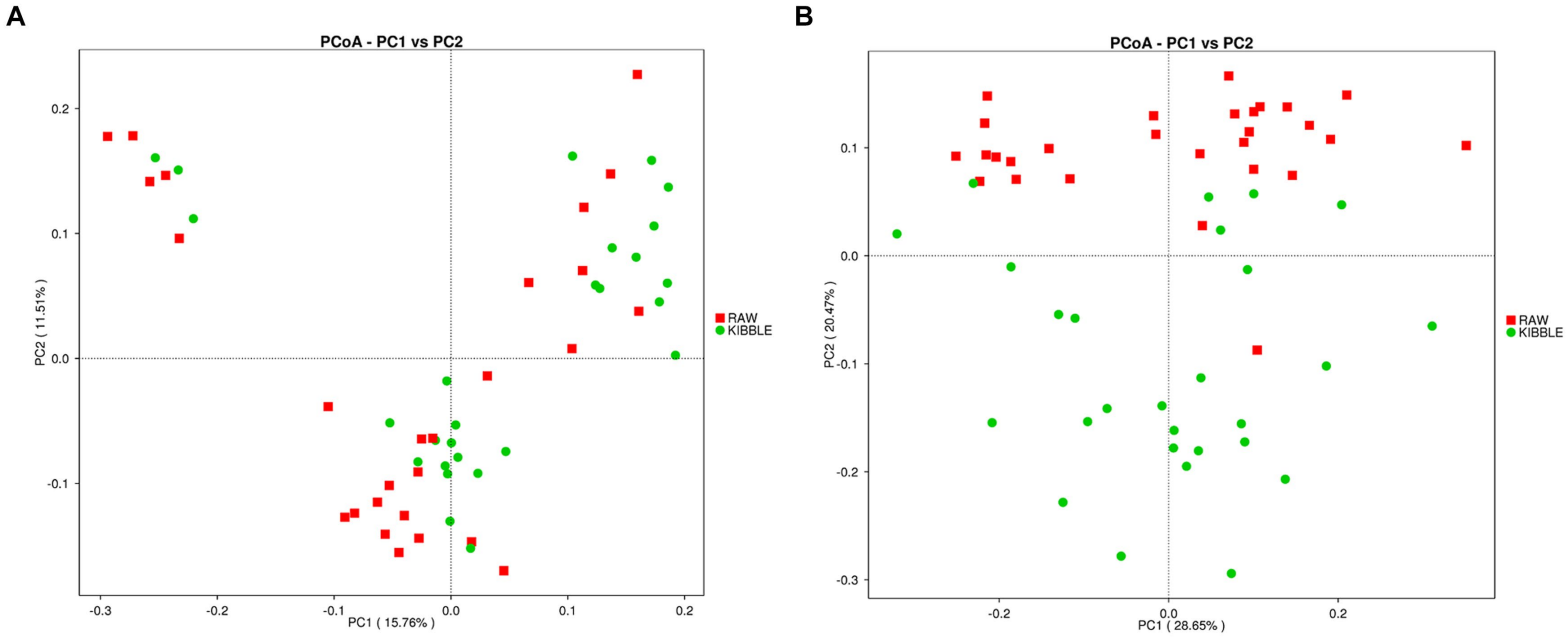
Beta diversity of the fecal bacterial community in individual dogs fed with kibble (KD) versus raw meat-based diets (RMBD). **(A)** Principal Coordinates Analysis (PCoA) of unweighted UniFrac distances and **(B)** PCoA of weighted UniFrac distances. Each node represents an individual dog. *n* = 27 for KD and *n* = 28 for RMBD.

**Figure 4:**
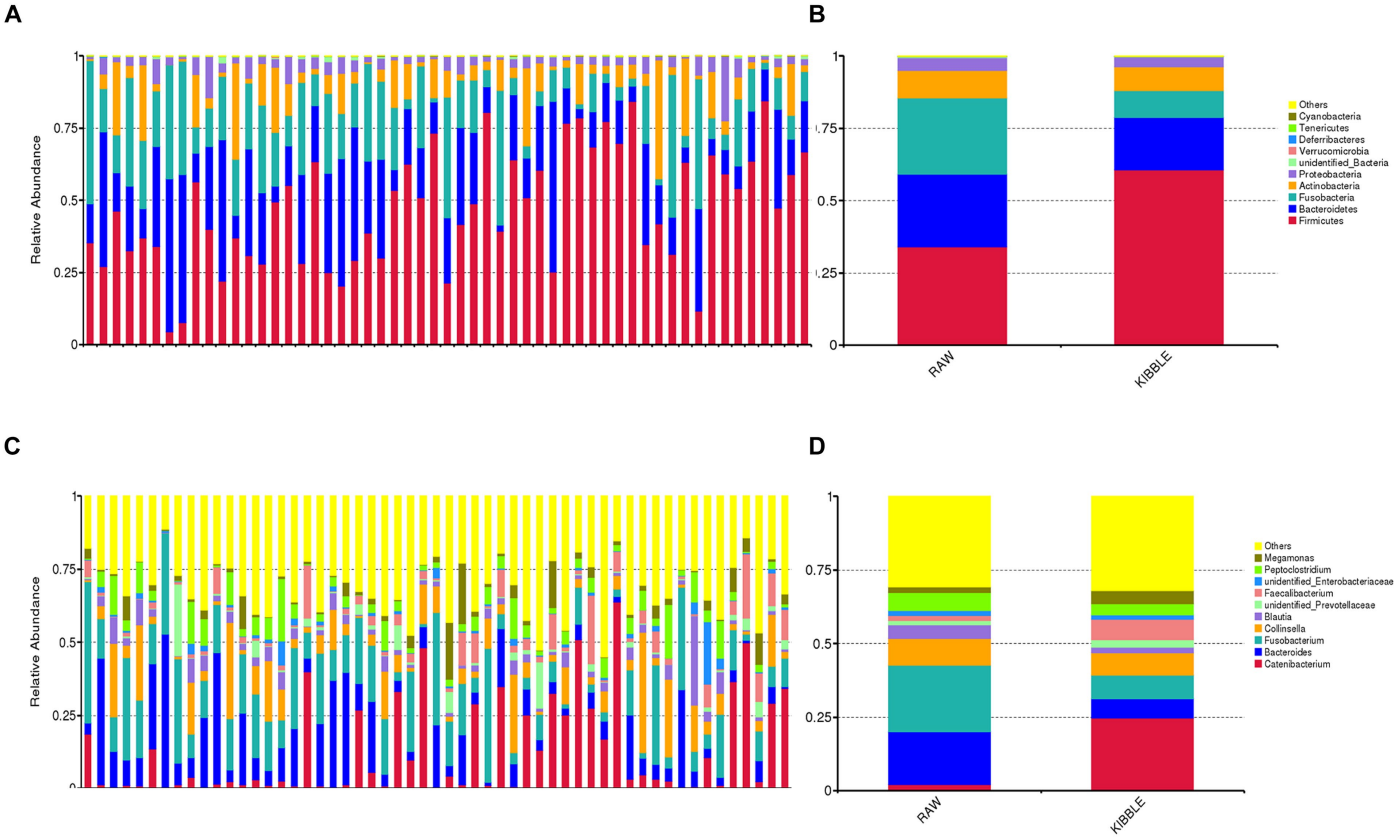
Fecal bacterial composition of dogs fed with kibble (KD) versus raw meat-based diets (RMBD) when data were analyzed based on individual dogs **(A,C)** or dietary groups **(B,D)**. The relative abundance of fecal bacterial community composition at **(A,B)** phylum and **(C,D)** genus levels. Only the top 10 phyla or genera are depicted for clarity. *n* = 27 for KD and *n* = 28 for RMBD.

**Figure 5:**
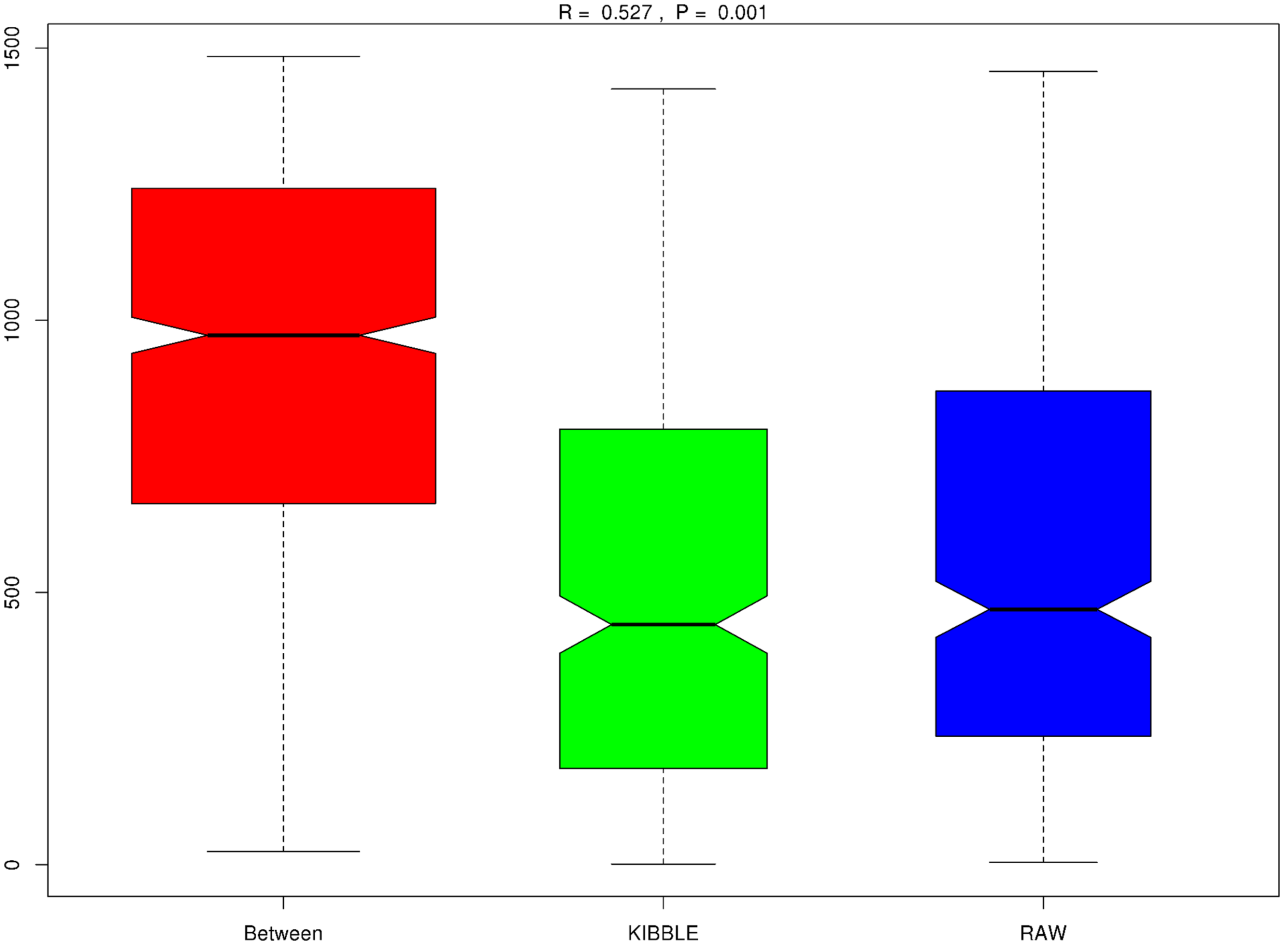
Beta diversity, Anisom results. Plotted with rank value on *Y*-axis and between groups or within groups on the *X*-axis. The positive *R*-value (*R* = 0.527) suggests the difference between groups is significantly greater than within group. *p* < 0.001.

**Figure 6:**
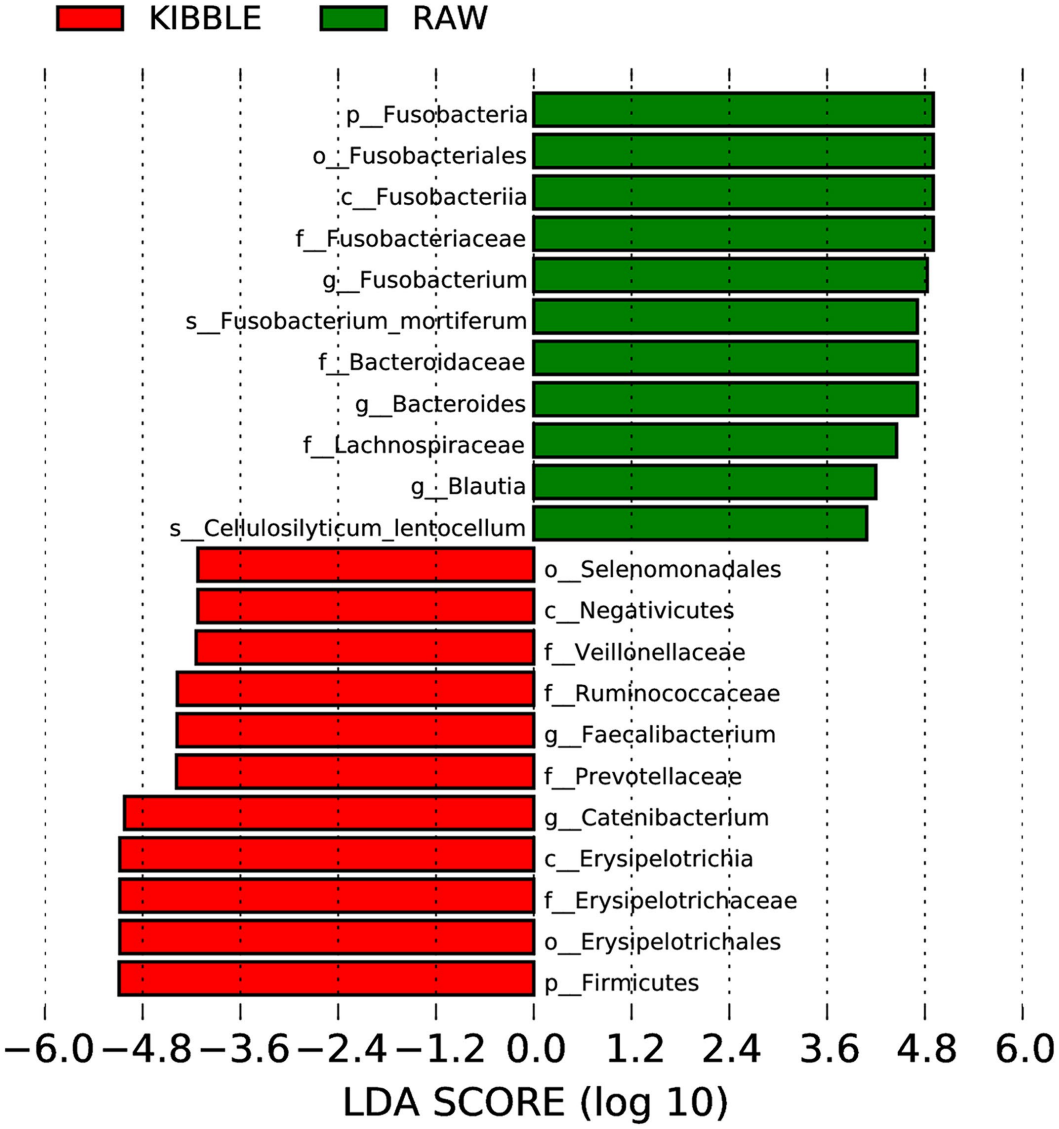
Histograms of linear discriminant analysis (LDA) with effect size (LEfSe) on fecal microbiota composition in dogs fed with kibble (KD) versus raw meat-based diets (RMBD). *n* = 27 for KD and *n* = 28 for RMBD.

Rarefaction curve analysis showed that all fecal samples analyzed reached a stable plateau at 20,000 with a 100,000 depth read and 300–500 OTU (Figures 1A,B) indicating that the sequence depth was adequate for capturing the species richness of the samples. The bacterial alpha diversity indices Chao1, observed species, Shannon, and Simpson are shown in Figures 2A–D. Chao1 and observed species did not differ in KD compared to RMBD (Figures 2A,B). However, Shannon and Simpson diversity indices were significantly different between the two diet groups (*p* < 0.05; Figures 2B–D), with RMBD showing greater diversity. Weighted PCoA, but not unweighted PCoA, showed a clear separation and clustering for fecal bacteria composition in dogs fed with KD versus RMBD (Figures 3A,B). Overall, the three main phyla in both KD and RMBD groups were Bacillota (Firmicutes), Bacteroidota, and Fusobacteriota (Figures 4A,B). Bacillota was the most abundant bacterial community at phylum level in both diets while their relative abundance was higher in KD (60.5%) than RMBD (34.0%) (Figures 4A,B), followed by Bacteroidota in KD (18.3%) and Fusobacteriota (26.3%) in RMBD. At genus level, the most abundant bacteria in the KD were *Catenibacterium* (24.7%), *Fusobacterium* (8.1%), *Collinsella* (7.5%) and *Faecalibacterium* (7.1%) (Figures 4C,D); while *Fusobacterium* was the most abundant genus in RMBD (22.6%), followed by *Bacteroides* (17.9%), and *Collinsera* (9.1%). Complete data on taxa, including p and q values can be found in Supplementary Tables S5–S10 and Supplementary Figures S1–S5. Beta diversity indices, Anosim (Figure 5), Adonis and AMOVA, all indicated a difference in species diversity between KD and RMBD (*p* < 0.001). Linear discriminant analysis (LDA) with effect size measurements (LEfSe) was used to assess the differences in gut bacterial abundances between KD and RMBD. Dogs fed RMBD had a higher proportion of p- Fusobacteria, o Fusobacteriales, c-Fusobacteriia, and f-Fusobacteriaceae families compare to KD fed dogs, while KD fed dogs had higher proportion of p-Firmicutes, o-Erysipelotrichales, f-Erysipelotrichaceae, c- Erysipelotrichia and g-Catenibacterium (LDA [log_10_] score > 2.0; Figure 6).

### Serum metabolomics

Serum metabolomics analysis resulted in identification of a total of 148 known metabolites with 75 being significantly different between KD and RMBD-fed dogs with age and BCS included in the regression analysis and 63 remaining significant after applying correction for multiple comparisons (Table 2; *p* ≤ 0.05).

**Table 2:** Serum metabolomics profile in dogs fed with kibble (KD) or raw meat-based diets (RMBD).

Metabolites	KD	RMBD	*P*-value- regression	Corrected *p*-value
Amino acids				
Valine	241,175 ± 46,975	407,682 ± 65,417	2.04E-10	4.35E-09
Isoleucine	174,848 ± 38,490	297,545 ± 58,740	1.72E-08	2.57E-07
Beta alanine	233 ± 67	321 ± 87	0.00027	0.0014
Glutamic acid	3,548 (2749–4,185)	3,362 (2797–3,877)	0.00044	0.0022
Tryptophan	184,998 ± 77,695	125,017 ± 59,761	0.0018	0.0072
Glutamine	227,537 ± 134,313	128,923 ± 102,342	0.0040	0.014
Phenylalanine	69,415 ± 17,134	57,213 ± 11,216	0.0042	0.014
Aspartic acid	4,087 (3421–4,788)	3,359 (2972–4,164)	0.014	0.042
Lysine	40,142 (28019–56,252)	51,703 (41971–63,051)	0.018	0.049
Leucine	565,888 ± 129,277	710,129 ± 148,391	0.023	0.056
Tyrosine	111,924 (88439–124,706)	91,489 (79088–103,304)	0.040	0.082
Amino acid derivatives and metabolites				
Alpha aminoadipic acid	293 (181–361)	747 (598–951) ± 306	3.13E-07	3.33E-06
2-aminobutyric acid (homoalanine)	28,814 (22060–36,994)	50,710 (43142–79,632)	1.02E-06	1.02E-05
2-hydroxybutanoic acid	77,690 (55167–93,990)	177,651 (115429–231,738)	1.11E-06	1.04E-05
Xanthurenic acid	91 ± 45	183 ± 72	4.15E-06	3.43E-05
Oxoproline	357,538 ± 61,551	287,172 ± 51,075	0.00010	0.00067
Cystine	11,680 ± 5,297	7,323 ± 4,124	0.0019	0.0072
*N*-acetylornithine	23,652 (21542–27,402)	19,930 (17509–22,001)	0.0041	0.014
Glutaric acid	240 (197–298)	209 (174–257)	0.0057	0.018
*Trans*-4-hydroxy-l-proline	27,207 ± 10,682	48,959 ± 23,657	0.016	0.045
*N*-acetyl-d-tryptophan	492 (429–659)	443 (305–540)	0.018	0.049
2-ketoisocaproic acid	28,429 ± 6,705	32,532 ± 5,857	0.019	0.049
Kynurenic acid	866 (462–1,160)	1931 (1244–2,389)	0.021	0.050
Kynurenine	730 (489–1,081)	403 (217–1,006)	0.028	0.065
Aminomalonate	8,184 (6085–12,552)	12,676 (8316–14,772)	0.031	0.07
Protein metabolism and urea cycle				
Urea	2,287,776 (2005977–2,600,273)	1,966,547 (915891–2,214,708)	0.045	0.090
Nucleic acids derivatives				
Pseudouridine	4,757 ± 131	4,308 ± 172	0.030	0.070
Vitamins				
Tocopherol alpha	32,791 (29236–37,439)	18,302 (12551–20,788)	5.89E-10	1.10E-08
Nicotinamide	742 (558–2026)	355 (235–577)	3.76E-05	0.00028
Tocopherol beta	56 (45–73)	251 (183–346)	0.00021	0.0012
Tocopherol gamma	175 (130–233)	247 (211–351)	0.02	0.05
Carbohydrates				
Raffinose	361 (265–414)	35 (26.5–46)	6.38E-14	3.17E-12
Mannose	48,786 ± 10,798	92,477 ± 21,363	8.89E-11	2.65E-09
Beta gentiobiose	793 (532–990)	80 (59–105)	1.86E-10	4.35E-09
Fructose	3,763 (2985–4,358)	2026 (1740–2,478)	0.00026	0.0014
Ribose	2,222 (2003–2,672)	1941 (1787–2,142)	0.0021	0.0077
Glucose	1,695,168 ± 209,437	1,402,373 ± 243,751	0.013	0.038
Sucrose	264 (203–345)	151 (101–220)	0.05	0.099
Carbohydrate derivatives				
Pinitol	4,305 (3006–5,021)	173 (120–225)	9.57E-16	1.43E-13
Galactinol	291 (217–356)	47 (40–52)	4.00E-14	2.98E-12
Saccharic acid	1,142 (846–1,500)	319 (242–430)	1.73E-11	6.43E-10
Ribonic acid	634 (522–737)	366 (319–427)	2.76E-08	3.74E-07
Arabitol	6,695 (5537–7,534)	3,022 (2588–3,592)	4.20E-08	5.22E-07
Threonic acid	4,591 (3732–5,133)	2,878 (2540–3,792)	3.39E-06	2.97E-05
Isothreonic acid	1,254 (1111–1,433)	1,023 (891–1,155)	4.15E-05	0.00029
Threitol	1,103 (1010–1,196)	778 (664–952)	8.64E-05	0.00058
Myo-inositol	45,605 (36480–51,744)	57,553 (51246–67,422)	0.0025	0.0088
Galactonic acid	267 (189–333)	205 (168–258)	0.0061	0.019
Levoglucosan	358 (313–447)	259 (196–315)	0.016	0.045
UDP-glucuronic acid	581 (434–1,037)	386 (329–608)	0.018	0.049
Carbohydrate metabolites				
Glucose-6-phosphate	350 ± 57	523 ± 102	2.39E-07	2.74E-06
Erythritol	4,070 ± 804	3,192 ± 707	0.00015	0.00095
2-hydroxyglutaric acid	361 (313–390)	565 (420–751)	0.00066	0.0030
Fumaric acid	5,429 ± 845	4,816 ± 821	0.019	0.049
Gluconic acid	289 ± 70	258 ± 63	0.036	0.078
Lipid metabolites				
Glycerol	131,128 (96356–146,532)	21,086 (178521–227,050)	0.0018	0.0072
Adipic acid	1834 (1287–2,306)	1,278 (1143–1,563)	0.00019	0.0011
Lipid derivatives and phenols				
4-hydroxybenzoate (benzoic acid)	354 ± 78	209 ± 50	9.44E-09	1.56E-07
4-hydroxybenzoic acid	724 (626–800)	511 (460–596)	0.00040	0.0020
3-phenyllactic acid	802 (629–1,079)	384 (311–604)	0.00096	0.0043
Phosphoethanolamine	439 ± 205	768 ± 354	0.0013	0.0055
4-hydroxymandelic acid	3,946 (3169–4,350)	2,819 (2417–3,487)	0.0013	0.0055
3-hydroxybutyric acid (beta hydroxybutyrate)	15,658 (12833–19,914)	24,903 (20688–32,633)	0.0030	0.010
4-hydroxyphenylacetic acid	545 (444–748)	1,001 (768–2,288)	0.041	0.084
Fatty acids and fatty acid metabolites				
Phytanic acid	170 (133–235)	368 (279–569)	1.23E-05	9.64E-05
Linoleic acid 18:3	12,228 (8145–17,673)	17,855 (11146–20,038)	0.034	0.076
Behenic acid 22:0	2,470 (2272–2,743)	2,113 (1971–2,709)	0.035	0.076
Arachidonic acid 20:4	9,876 ± 3,674	12,657 ± 2,852	0.016	0.045
Heptadecanoic acid (margaric acid 17:0)	15,798 ± 4,398	12,425 ± 3,759	0.0010	0.0045
2-hydroxyhexanoic acid	1,548 (1386–2,351)	2,187 (1678–2,681)	0.020	0.050
Arachidic acid (icosanoic acid 20:0)	6,566 ± 2,518	5,419 ± 2006	0.035	0.076
Maleic acid	373 (346–447)	323 (287–366)	0.038	0.081
Myristic acid 14:0	3,951 (3075–4,957)	3,464 (2651–3,958)	0.038	0.081
Monoamines				
*N*-acetyl-5-hydroxytryptamine	168,116 ± 41,644	135,095 ± 28,715	0.00051	0.0024
Microbiome metabolism				
Indole-3-propionic acid	3,628 (1848–5,588)	862 (517–1,204)	0.0022	0.0079

Results listed as mean ± SD. If data did not pass normality testing (Shapiro–Wilks), results listed as median (IQR). Serum metabolite concentration was compared by multiple linear regression, with diet group, age and BCS as independent variables. *P-*values were corrected for multiple comparisons using Benjamini–Hochberg procedure to control False Discovery Rate.

**Figure 7:**
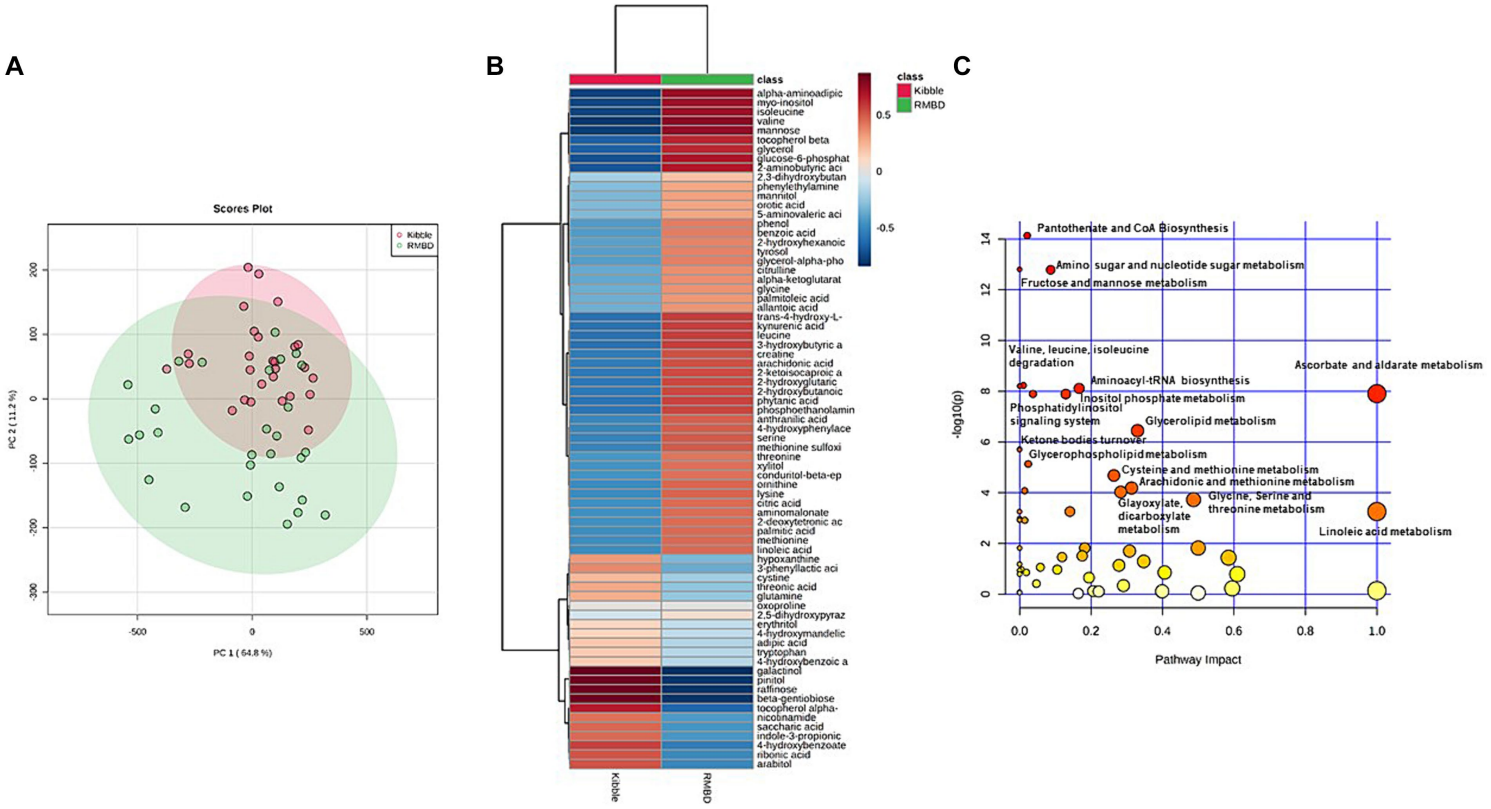
Serum metabolites of dogs fed with kibble (KD) versus raw meat-based diets (RMBD) displayed by score plots of principal component analysis (PCA), heat map, and the pathway analysis map. **(A)** PCA score plot of dogs’ serum metabolites. Dogs are shown by individual circles. **(B)** Hierarchical clustering of all significantly different serum metabolites between RMBD and KD groups. Columns indicate the dietary groups (RMBD vs. KD). The red color indicates the high abundance and blue color indicates the low abundance. **(C)** The pathway analysis map for the identified metabolites in the serum. The circles demonstrate the metabolic pathways. The scores for each circle were obtained from topology analysis with pathway impact for *x*-axis and analysis of the pathway enrichment for *y*-axis. The circle size reflects its impact value indicative of greater pathway impact for the large size circles. The circle color is based on its *p*-value meaning the darker color circles show more significant metabolite modifications and greater pathway enrichment. *n* = 27 for KD and *n* = 28 for RMBD.

The PCA revealed a separation in metabolites for the majority of dogs in KD and RMBD groups. PC1 is indicative of 64.8% variation in metabolites changes among samples and PC2 explains 11.2% of the variation (Figure 7A). Hierarchical clustering heat map showed KD and RMBD groups had differentially expressed metabolites (Figure 7B). The metabolic pathway enrichment analysis showed that pantothenate and CoA biosynthesis, amino sugar and nucleotide sugar metabolism, fructose and mannose metabolism, ascorbate and aldarate metabolism, valine, leucine, and isoleucine degradation, aminoacyl-tRNA biosynthesis, inositol phosphate metabolism, phosphatidyl inositol signaling metabolism and glycerolipid metabolism pathways were different between KD and RMBD groups (Figure 7C). Amino acids, as well as amino acid derivatives and metabolites were significantly different between the two dietary groups. Moreover, dietary groups showed differential changes in carbohydrates, carbohydrates derivatives and metabolism, lipid metabolites and derivatives, fatty acids and vitamin metabolites. The list of identified metabolites that were not significantly different between KD and RMBD are provided in Supplementary Table S11.

### Inflammatory markers

Fecal IAP, IgG, and IgA were significantly higher in RMBD dogs compared to KD dogs (*p* < 0.0004, *p* < 0.0006, *p* < 0.0002 respectively), when the effect of age and BCS were included in the analysis (Table 3). Serum CRP, galectin, sRAGE, haptoglobin, and IgG were similar in dogs fed either diet (Table 3).

**Table 3:** Inflammatory markers by feed group.

Inflammatory biomarker	Sample type	KD, *n* = 27		RMBD, *n* = 28		*P-*value
		Mean or median	95% CI	Mean or median	95% CI	
CRP (mg/L)	Serum	2.2	1.8–2.7	2.4	1.9–2.9	0.84
IgG^*^	Serum	317.6	256–354	339	296–377	0.26
sRAGE^*^	Serum	0.45^**^	0.39–0.57	0.4	0.38–0.47	0.69
Galectin	Plasma	55.3	47.4–61.3	49.2	44.4, 54.0	0.89
Haptoglobin^*^	Plasma	0.38	0.30–0.56	0.47	0.32–0.73	0.99
IAP^*^	Feces	7.0	3.2–10.5	20.7	13.7–32.4	<0.001
IgA^*^	Feces	104	63–186	234	132–381	0.0035
IgG^*^	Feces	0.28	0.085–0.66	0.74	0.45–1.37	<0.001

Mean or median values with 95% confidence interval (CI) for inflammatory markers in dogs fed RMBD or KD. *P-*values were calculated by regression analysis for the effect of diet on inflammatory markers, adjusting for age and BCS. Data was log transformed as needed. ^*^Data log transformed for analysis; median value presented. ^**^n = 25. KD, kibble diet; RMBD, raw meat-based diet; CI, confidence interval; CRP, c-reactive protein; IgG, immunoglobulin G; sRAGE, soluble receptor for advanced glycation end-products; IAP, intestinal alkaline phosphatase; IgA, immunoglobulin A.

## Discussion

As expected, numerous differences were apparent in the fecal microbiota and the metabolome of dogs fed markedly different diets. Despite this, serum inflammatory markers were similar between the two groups, although intestinal markers of inflammation were strikingly different. It is impossible to determine whether diet ingredient composition, processing, or both contributed to differences observed. Furthermore, it is unclear if any of the observed changes were beneficial or detrimental as only healthy dogs were included in this study. Ideally, a large cohort of dogs should be followed over time to determine how these findings associate with clinically relevant disease risk. Additionally, a cross over design which exposed dogs to both diets would have been ideal to isolate diet effects on intestinal inflammation.

Diet composition, including macronutrient composition, ingredient source and processing, has a substantial impact on gut microbiota. Regardless of diet, the three main phyla in both KD and RMBD diets were Bacillota, Bacteriodota, and Fusobacteriota, which agrees with what has been observed by others (47–51). Like other studies, dogs fed RMBD had higher Fusobacteriota and Bacteriodota in their feces, while Bacillota, was more abundant in dogs fed a KD (47–51). Although high numbers of Fusobacterium have been associated with diseases such as colon cancer and IBD in humans (52), it appears to be a consistent finding in the healthy dog (47, 53). Bacillota, which metabolize plant polysaccharides, have been shown to increase in omnivores (both dogs and people) when the diet is shifted toward plant-based ingredients, as was true for KD dogs (48, 49, 54–57). Similarly, the abundance of *Bacteroides* increases in humans consuming an animal-based diet, compared to plant based (58, 59). However, the current study did not agree with others which have shown differences in *Faecalibacterium, Prevotella, Actinobacteria* and notably *Clostridium* in the feces of RMBD compared to KD dogs (54, 60). *Clostridiaceae* have been shown to positively correlate with protein content and digestibility, and to be present in cat feces that receive higher amount of protein in their diet (49). The family of *Erysipelotrichaceae* was in greater abundance in the feces of kibble fed dogs, similar to what was observed by Bermingham et al. (60). Furthermore, Bermingham et al. (60) reported a positive correlation between *Erysipelotrichaceae* and carbohydrate digestion. At the genus level, g-*Catenibacterium* was the most prevalent bacteria in KD dogs, which is likely reflective of the difference in diet ingredients. This bacterium utilizes many sugars as the substrates of fermentation and produce SCFA.

In line with findings of Castañeda et al. (47) and Kim et al. (61) Shannon and Simpson diversity indices of alpha diversity was greater in dogs eating RMBD. Although increased diversity is generally considered to reflect improved intestinal and systemic health (62–65), this is likely an oversimplification as many factors can affect alpha diversity, including breed and body condition, both of which differed in the two feeding groups of this study (66–68). Weighted PCoA as an index of β-diversity also showed a clear separation in fecal microbiota between two groups as reported previously by others (47, 61). A clear separation for weighted PCoA, but not unweighted PCoA between RMBD and KD dogs is suggestive of the differences in abundance of bacteria rather than their presence/absence between these two groups.

After adjustment for BCS and age, dogs in our study showed differences in 63 serum metabolites, primarily in those related to carbohydrate, fat, and protein metabolism. Most observed differences in metabolites are likely explained by differences in macronutrient composition of the diets. The inclusion of plant products (rice, wheat, corn, and soybean meal) in KD increased the amount of total carbohydrates (sugars, starch, and fiber) while RMBD had more protein and fat from solely animal sources (ground poultry, beef, salmon, egg, and beef organ). To date, only two studies have reported the metabolomics of raw fed dogs (48, 69). Evaluation of the fecal metabolome in dogs fed Bones and Raw Food (BARF) diets and commercial diets, revealed only minor difference as isomaltose, 4-aminobutyric acid (GABA) and 4-hydroxybutryic acid (GHB) were the only metabolites that differed between the groups (69). Our investigation did not evaluate the fecal metabolome which may have proven useful for comparison.

Overall metabolites of protein metabolism, including amino acids, their metabolites and urea differed between groups. While diet composition differed, feeding rates resulted in a relatively similar daily protein intake between the two groups, (RMBD = 3.6 g/kg bwt/day; KD = 3.2 g/kg bwt/day). However, protein source (animal origins only, versus animal plus plant protein sources) as well as processing (grinding and freezing only versus high temperature/pressure) did differ between diets. Amino acid (AA) intake on DM/kg bwt basis was not consistently indicative of what was seen in plasma, with higher dietary intake not always paralleling higher serum concentrations. Alterations in plasma amino acids compared to dietary source could be attributed to alterations in protein synthesis or catabolism or the digestibility and bioavailability of the feed. Previously, others have shown that rendering protein products, as well as processing decreases crude protein (CP) and AA digestibility. Digestibility of protein in raw meat diets has been shown to be quite high, reported at 95–98% (55, 69–71).

As expected, the largest variation in metabolomics was in carbohydrate metabolism as identified through pathway analysis. The KD had 32.3% starch, with KD fed dogs consuming 3 g starch/kg/bwt daily, while starch consumption was negligible (<1%) in RMBD due to the non-inclusion of plant sources in their diet. KD fed dogs were higher in raffinose, beta gentiobiose (a byproduct of glucose caramelization), pinitol, galactinol, glucose, sucrose, ribose, and fructose all of which were present in higher quantities in the diet due to plant products.

We did not see many differences in lipid profiles despite the dogs being fed substantially different quantities of fat/kg bwt. Serum metabolites of RMBD dogs in our study were higher only in linoleic and arachidonic acid, while Puurunen et al. (69) found raw fed dogs to be higher in linoleic, stearic, and total saturated fatty acids. A reliance of beta oxidation from fatty acids for energy production in the absence of carbohydrates is evident in the RMBD dogs through the elevation of the ketone body, beta hydroxybutyrate. Ketogenic diets fed to dogs have been shown to alter phosphatidylcholine and acylcarnitine metabolites (72). Unfortunately, the metabolomics performed in the current study was selected to identify primary metabolites, and thus did not target identification of more complex lipids.

Beneficial antioxidant and anti-inflammatory metabolites might contribute to improved health. Those that were higher in the KD dogs included alpha tocopherols and niacin, presumably due to direct inclusion of these nutrients in kibble diets. Additionally, KD dogs consumed 3-fold more zinc, an important co-factor for many enzymes in the antioxidant defense system. RMBD dogs were higher in beta and gamma tocopherols as well as essential fatty acids, arachidonic acid, and linoleic acid. In theory, both fatty acids may contribute to improved skin health and immune function, two health benefits which advocates assert are associated with feeding dogs a RMBD.

Despite numerous differences in serum metabolites including those with anti-inflammatory and antioxidant functions, serum markers of inflammation did not differ between feed groups. Two acute phase proteins, (CRP and haptoglobin) and serum IgG were selected to broadly assess inflammation. Previous data from the same group of animals found a lower serum globulin concentration, a lower serum alkaline phosphatase activity and a greater lymphocyte count in dogs fed raw when corrected for age and BCS (46). Despite these previous indicators, we found no evidence that either diet was associated with systemic inflammation. It is conceivable that the selected markers were not sensitive enough to discriminate differences in clinically healthy animals. Larger studies with additional markers including functional assays might help answer these questions.

We also anticipated that sRAGE and galactin-3, known AGE binding proteins, would be elevated in KD dogs as processed food has been associated with high dietary AGE levels (11, 72). Although to the author’s knowledge there is no published data directly comparing AGEs in raw to kibble diet, Palaseweenun et al. (13) showed increased urinary AGEs in dogs fed kibble compared to raw fed dogs. Furthermore, it is known that processed dog food has 122 times more AGEs than human food (11). However, in the current study, neither sRAGE nor galactin-3 differed between groups. In contrast, dogs fed a dry food had higher levels of glycoprotein acetyls, representing glycated proteins within the blood associated with systemic inflammation (69). RAGE is a pattern recognition receptor which recognizes pathogen associated molecular patterns and endogenous molecular structures expressed at sites of inflammation (73–75). sRAGE is a variant of RAGE and functions as an anti-inflammatory decoy, sequestering ligands of RAGE to eliminate proinflammatory signals (76). Using a ratio of AGE:sRAGE may have been more useful in detecting inflammation (76). Our group did not measure serum AGEs, a limitation for this study in evaluating for inflammatory risk.

While systemic markers of inflammation did not differ between the groups, fecal anti-inflammatory markers, including fecal IgA, IgG, and IAP, were significantly higher in RMBD than KD dogs. We theorize these increases in RMBD fed dogs may reflect improved gastrointestinal homeostasis and immune function as well as increased feed digestibility. The marked degree of significance in these markers is an encouraging insight supporting the anecdotal evidence of improved health outcomes in raw fed dogs. IAP, an isoenzyme of alkaline phosphatase, is produced exclusively in the intestinal tract by villus-associated enterocytes and plays a protective role by detoxifying bacterial LPS and upregulating the expression of intestinal tight junction proteins (40, 77–80). Decreased IAP expression is associated with inflammatory diseases (45) and plays a significant role in the development of canine chronic enteropathies (45); dogs with chronic enteropathies had reduced IAP expression and reduced lipopolysaccharide (LPS) dephosphorylation activity (40). Increased IAP levels in RMBD fed dogs could reflect improved gut luminal detoxification and potentially reduced susceptibility to inflammatory conditions through impact on the microbiome and reduced intestinal inflammation and permeability.

Both age and diet have been shown to influence intestinal IgA secretion with increasing age diminishing antigen specific mucosal IgA responses in many species (81, 82). In our study, RMBD fed dogs were older, yet fecal IgA was significantly increased compared to KD dogs. Highly digestible foods have been shown to increase fecal IgA in both adult and geriatric dogs (83). Therefore, it is possible, that the greater digestibility of RMBD may account for the increase in fecal IgA levels. Whether or not this increase in IgA has physiological importance is unknown.

The role of higher concentration of fecal IgA and IgG in RMBD dogs is less definitive as it may be indicative of improved gastrointestinal immune function or alternatively, may indicate chronic low-level exposure to inflammatory enteric pathogens. IgA is the most abundant antibody isotope in the body and is essential for mucosal immunity and steady-state health in the gastrointestinal tract responding to pathogens, commensals and dietary allergens (84). Secretory IgA offers both immune protection, guarding against pathogenic microorganism colonization and invasion as well as immune tolerance and confinement of commensal flora to the intestinal lumen (84, 85). Secretory IgA deficiency has been shown to increase the risk of inflammatory, autoimmune, allergic, neoplastic and infectious disease (84). Intestinal and fecal IgA was lower in dogs with inflammatory bowel disease compared to healthy dogs (86). In contrast, people with IBD, celiac disease or Crohn’s disease have been shown to have increased total intestinal IgA and IgG (87).

The authors speculated that differences in fecal markers of inflammation would correlate with systemic markers of inflammation. However, this was not the case. This could reflect lack of sensitivity of the biomarkers. Enrolled dogs met strict inclusion criteria designed to select only healthy animals, therefore the study design may have served to screen out animals with early systemic inflammation of a sufficient magnitude to be detected by markers previously used to identify dogs with overt inflammatory disease.

Alterations of the gastrointestinal microbiome have the potential to impact tract health, as reflected through markers of inflammation. This is the first study to look at the impact of feeding practices directly on gut anti-inflammatory markers. In the current study, fecal IAP, IgA, and IgG levels were all higher in the RMBD group compared to the KD group. While the increase in immunoglobulins might reflect a healthier microenvironment or conversely a response to chronic enteric pathogen exposure, IAP is a critical enzyme in intestinal microbial homeostasis and barrier function and an increase in IAP activity positively correlates with markers of intestinal health.

## Conclusion

Feeding minimally processed diets higher in protein with fewer plant-sourced carbohydrates changed microbial populations as well as serum metabolites.

Our study is the first to report that RMBD diets are associated with increased fecal IAP, IgA, and IgG. Further work is necessary to clearly delineate the impact of these findings on gastrointestinal homeostasis and immune function. as well as to understand the long-term effects of these diets on canine health.

## Data availability statement

The data presented in the study are deposited in the BioProject repository, accession number BioProject ID PRJNA1089542.

## Ethics statement

The animal studies were approved by Oklahoma State University’s Institutional Animal Care and Use Committee (ACUP# VM-18-26). The studies were conducted in accordance with the local legislation and institutional requirements. Written informed consent was obtained from the owners for the participation of their animals in this study.

## Author contributions

KH: Conceptualization, Funding acquisition, Investigation, Methodology, Supervision, Writing – original draft, Writing – review & editing. LS: Conceptualization, Funding acquisition, Investigation, Methodology, Supervision, Writing – original draft, Writing – review & editing. UD: Writing - review & editing. AP: Conceptualization, Investigation, Methodology, Writing – original draft, Writing – review & editing. PR: Writing – original draft, Writing – review & editing, Formal analysis. PG: Formal analysis, Writing – original draft, Writing – review & editing. EW: Methodology, Writing – original draft, Writing – review & editing. DM: Conceptualization, Data curation, Funding acquisition, Investigation, Methodology, Project administration, Supervision, Writing – original draft, Writing – review & editing.

## Supplementary material

SUPPLEMENTARY FIGURE S1 *T*-test of between group variation in phylum. The left panel shows the species that differ significantly between groups. Each bar represents the mean value of the abundance in each group of the species showing significant difference between group. The right panel is the confidential interval of between group variation, with the bars indicating the 95% confidential interval. The center of the circle stands for the difference of the mean value. The color of the circle is in agree with the group whose mean value is higher. The *p*-value for the between group variation is on the right.

SUPPLEMENTARY FIGURE S2 T-test of between group variation in class. The left panel shows the species that differ significantly between groups. Each bar represents the mean value of the abundance in each group of the species showing significant difference between group. The right panel is the confidential interval of between group variation, with the bars indicating the 95% confidential interval. The center of the circle stands for the difference of the mean value. The color of the circle is in agree with the group whose mean value is higher. The *p*-value for the between group variation is on the right.

SUPPLEMENTARY FIGURE S3 *T*-test of between group variation in order. The left panel shows the species that differ significantly between groups. Each bar represents the mean value of the abundance in each group of the species showing significant difference between group. The right panel is the confidential interval of between group variation, with the bars indicating the 95% confidential interval. The center of the circle stands for the difference of the mean value. The color of the circle is in agree with the group whose mean value is higher. The p-value for the between group variation is on the right

SUPPLEMENTARY FIGURE S4 T-test of between group variation in family. The left panel shows the species that differ significantly between groups. Each bar represents the mean value of the abundance in each group of the species showing significant difference between group. The right panel is the confidential interval of between group variation, with the bars indicating the 95% confidential interval. The center of the circle stands for the difference of the mean value. The color of the circle is in agree with the group whose mean value is higher. The p-value for the between group variation is on the right.

SUPPLEMENTARY FIGURE S5 T-test of between group variation in genus. The left panel shows the species that differ significantly between groups. Each bar represents the mean value of the abundance in each group of the species showing significant difference between group. The right panel is the confidential interval of between group variation, with the bars indicating the 95% confidential interval. The center of the circle stands for the difference of the mean value. The color of the circle is in agree with the group whose mean value is higher. The *p*-value for the between group variation is on the right.

SUPPLEMENTARY FIGURE S6 T-test of between group variation in species. The left panel shows the species that differ significantly between groups. Each bar represents the mean value of the abundance in each group of the species showing significant difference between group. The right panel is the confidential interval of between group variation, with the bars indicating the 95% confidential interval. The center of the circle stands for the difference of the mean value. The color of the circle is in agree with the group whose mean value is higher. The p-value for the between group variation is on the right.

## References

[1] Kazimierska K Biel W Witkowicz R Karakulska J Stachurskah X. Evaluation of nutritional value and microbiological safety in commercial dog food. Vet Res Commun. (2021) 45:111–28. doi: 10.1007/s11259-021-09791-6 33903989 PMC8373756

[2] Pigłowski M. Pathogenic and non-pathogenic microorganisms in the rapid alert system for food and feed. Int J Environ Res Public Health. (2019) 16:477. doi: 10.3390/ijerph16030477 30736316 PMC6388125

[3] Behravesh CB Ferraro A Deasy M Dato V Moll M Sandt C. Human Salmonella infections linked to contaminated dry dog and cat food, 2006-2008. Pediatrics. (2010) 126:477–83. doi: 10.1542/peds.2009-3273 20696725

[4] Skinner CG Thomas JD Osterloh JD. Melamine toxicity. J Med Toxicol. (2010) 6:50–5. doi: 10.1007/s13181-010-0038-1 20195812 PMC3550444

[5] Dobson RLM Motlagh S Quijano M Cambron RT Baker TR Pullen AM. Identification and characterization of toxicity of contaminants in pet food leading to an outbreak of renal toxicity in cats and dogs. Toxicol Sci. (2008) 106:251–62. doi: 10.1093/toxsci/kfn160 18689873

[6] Boermans HJ Leung MCK. Mycotoxins and the pet food industry: toxicological evidence and risk assessment. Inter J Food Microbiol. (2007) 119:95–102. doi: 10.1016/j.ijfoodmicro.2007.07.063 17889389

[7] Bischoff K Rumbeiha WK. Pet food recalls and pet food contaminants in small animals. Vet Clin North Am Small Anim Pract. (2018) 48:917–31. doi: 10.1016/j.cvsm.2018.07.005 30173926

[8] Bakke AM Wood J Salt C Allaway D Gilham M Kuhlman G. Responses in randomised groups of healthy, adult Labrador retrievers fed grain-free diets with high legume inclusion for 30 days display commonalities with dogs with suspected dilated cardiomyopathy. BMC Vet Res. (2022) 18:1–17. doi: 10.1186/s12917-022-03264-x 35581582 PMC9112553

[9] Kaplan JL Stern JA Fascetti AJ Larsen JA Skolnik H Peddle GD. Taurine deficiency and dilated cardiomyopathy in Golden retrievers fed commercial diets. PLoS One. (2018) 13:–e0209112. doi: 10.1371/journal.pone.0209112 PMC6292607 30543707

[10] Quest BW Leach SB Garimella S Konie A Clark SD. Incidence of canine dilated cardiomyopathy diagnosed at referral institutes and grain-free pet food store sales: a retrospective survey. Front Anim Sci. (2022) 3:846227. doi: 10.3389/fanim.2022.846227

[11] Williams PA Hodgkinson SM Rutherfurd SM Hendriks WH. Lysine content in canine diets can be severely heat damaged. J Nutr. (2006) 136:1998S–2000S. doi: 10.1093/jn/136.7.1998S 16772478

[12] van Rooijen C Bosch G van der Poel AF Wierenga PA Alesander L Henriks WH. Quantitation of Maillard reaction products in commercially available pet foods. J Agric Food Chem. (2014) 62:8883–91. doi: 10.1021/jf502064h 25088431

[13] Palaseweenun P Hagen-Plantinga EA Schonewille JT Koop G Butre C Jonathan M. Urinary excretion of advanced glycation end products in dogs and cats. J Anim Physiol Anim Nutr. (2021) 105:149–56. doi: 10.1111/jpn.13347 PMC7818435 32279406

[14] Zhang Q Wang Y, L. Fu L. Dietary advanced glycation end-products: perspectives linking food processing with health implications. Compr Rev Food Sci Food Saf (2020) 19:2559–2587. doi: 10.1111/1541-4337.12593 33336972

[15] Costa JL Pedreira RS Gomes AC Restan AZ Vasconcellos RS Loureiro BA. Concentration of synthetic antioxidants and peroxide value of commercial dry pet foods. Anim Feed Sci Technol. (2022) 294:115499. doi: 10.1016/j.anifeedsci.2022.115499

[16] Vuori KA Hemida M Moore R Salin S Rosendahl S Anturaniemi J. The effect of puppyhood and adolescent diet on the incidence of chronic enteropathy in dogs later in life. Sci Rep. (2023) 13:1830. doi: 10.1038/s41598-023-27866-z 36759678 PMC9911636

[17] Schlesinger DP Joffe DJ. Raw food diets in companion animals: a critical review. Can Vet J. (2011) 52:50–4. 21461207 PMC3003575

[18] Dinallo GK Poplarski JA Van Deventer GM Eirmann LA Wakshlag JJ. A survey of feeding, activity, supplement use and energy consumption in north American agility dogs. J Nutr Sci. (2017) 6:e45. doi: 10.1017/jns.2017.44 29152249 PMC5672316

[19] Hoummady S Fantinati M Maso D Bynens A Banuls D Santos NR. Comparison of canine owner profile according to food choice: An online preliminary survey in France. BMC Vet Res. (2022) 18:163. doi: 10.1186/s12917-022-03258-9 35509073 PMC9066993

[20] Lenz J Joffe D Kauffman M Ahang Y LeJeune J. Perceptions, practices, and consequences associated with foodborne pathogens and the feeding of raw meat to dogs. Can Vet J. (2009) 50:637–43. 19721784 PMC2684052

[21] Freeman LM Chandler ML Hamper BA Weeth LP. Current knowledge about the risks and benefits of raw meat-based diets for dogs and cats. J Am Vet Med Assoc. (2013) 243:1549–58. doi: 10.2460/javma.243.11.1549 24261804

[22] Freitas AR Finisterra L Tedim AP Duarte B Novais C Peixe L. From the ESCMID study group on food- and water-borne infections (EFWISG). Linezolid- and multidrug-resistant enterococci in raw commercial dog food, Europe, 2019-2020. Emerg Infect Dis. (2021) 27:2221–4. doi: 10.3201/eid2708.204933 34287135 PMC8314808

[23] Nemser S Doran T Grabenstein M McConnell T McGrath T Pamboukian R. Investigation of Listeria, Salmonella, and toxigenic *Escherichia coli* in various pet foods. Foodborne Pathog Dis. (2014) 11:706–9. doi: 10.1089/fpd.2014.1748 24824368 PMC4152787

[24] Van Bree FP Bokken GC Mineur R Franssen F Opsteegh M van der Giessen JW. Zoonotic bacteria and parasites found in raw meat-based diets for cats and dogs. Vet Rec. (2018) 182:50. doi: 10.1136/vr.104535 29326391

[25] LeJeune JT Hancock DD. Public health concerns associated with feeding raw meat diets to dogs. J Am Vet Med Assoc. (2001) 19:1222–5. doi: 10.2460/javma.2001.219.1222 11697364

[26] Joffe DJ Schlesingher DP. Preliminary assessment of the risk of Salmonella infection in dogs fed raw chicken diets. Can Vet J. (2011) 43:441–2. PMC339295 12058569

[27] Weese JS Rousseau J Arroyo L. Bacteriological evaluation of commercial canine and feline raw diets. Can Vet J. (2005) 46:513–6. 16048011 PMC1140397

[28] Strohmeyer RA Morley PS Hyatt DR Dargatz DA Scorza AV Lappin MR. Evaluation of bacterial and protozoal contamination of commercially available raw meat diets for dogs. J Am Vet Med Assoc. (2006) 228:537–42. doi: 10.2460/javma.228.4.537 16478425

[29] Lefebvre SL Reid-Smith R Boerlin P Weese JS. Evaluation of the risks of shedding salmonellae and other potential pathogens by therapy dogs few raw diets in Ontario and Alberta. Zoonoses Public Health. (2008) 55:470–80. doi: 10.1111/j.1863-2378.2008.01145.x 18811908

[30] Finley R Reid-Smith R Weese JS. Human health implications of *Salmonella*-contaminated natural pet treats and raw pet food. Clin Infect Dis. (2006) 42:686–91. doi: 10.1086/500211 16447116

[31] Anturaniemi J Barrouin-Melo SM Zaldivar-López S Sinkko H Hielm-Björkman A. Owners’ perception of acquiring infections through raw pet food: a comprehensive internet-based survey. Vet Rec. (2019) 185:658–8. doi: 10.1136/vr.105122 31427409 PMC6952838

[32] Hajek V Zablotski Y Kölle P. Computer-aided ration calculation (diet check Munich©) versus blood profile in raw fed privately owned dogs. J Anim Physiol Anim Nutr. (2022) 106:345–54. doi: 10.1111/jpn.13601 34236742

[33] Pilla R Suchodolski JS. The role of the canine gut microbiome and metabolome in health and gastrointestinal disease. Front Vet Sci. (2019) 6:498. doi: 10.3389/fvets.2019.00498 31993446 PMC6971114

[34] Mondo E Marliani G Accorsi PA Cocchi M Di Leone A. Role of gut microbiota in dog and cat's health and diseases. Open Vet J. (2019) 9:253–8. doi: 10.4314/ovj.v9i3.10 31998619 PMC6794400

[35] Tizard IR Jones SW. The microbiota regulates immunity and immunologic diseases in dogs and cats. Vet Clin North Am Small Anim Pract. (2018) 48:307–22. doi: 10.1016/j.cvsm.2017.10.008 29198905

[36] Marsella R. De Benedetto A. Atopic dermatitis in animals and people: an update and comparative review. Vet Sci (2017) 4:37. doi: 10.3390/vetsci4030037 29056696 PMC5644664

[37] Casén C Vebo HC Sekelja M Hegge FT Karlsson MK Ciemniejewska E. Deviations in human gut microbiota: a novel diagnostic test for determining dysbiosis in patients with IBS or IBD. Aliment Pharmacol Ther. (2015) 42:71–83. doi: 10.1111/apt.13236 25973666 PMC5029765

[38] Craig JM. Atopic dermatitis and the intestinal microbiota in humans and dogs. Vet Med Sci. (2016) 2:95–105. doi: 10.1002/vms3.24 29067183 PMC5645856

[39] Mulder IE Schmidt B Stokes CR Lewis M Bailery M Aminove RI. Environmentally-acquired bacteria influence microbial diversity and natural innate immune responses at gut surfaces. BMC Biol. (2009) 7:79. doi: 10.1186/1741-7007-7-79 19930542 PMC2785767

[40] Ide K Kato K Sawa Y Hayaski A Rakizawa R Nishifuji K. Comparison of the expression, activity, and fecal concentration of intestinal alkaline phosphatase between healthy dogs and dogs with chronic enteropathy. Am J Vet Res. (2016) 77:721–9. doi: 10.2460/ajvr.77.7.721 27347825

[41] Lassenius MI Fogarty CL Blaut M Haimila K Rittinen L Paju A. Intestinal alkaline phosphatase at the crossroad of intestinal health and disease–a putative role in type 1 diabetes. J Intern Med. (2017) 281:586–600. doi: 10.1111/joim.12607 28393441

[42] Cabrera-García AI Suchodolski JS Steiner JM Heilmann RM. Association between serum soluble receptor for advanced glycation end-products (RAGE) deficiency and severity of clinicopathologic evidence of canine chronic inflammatory enteropathy. J Vet Diagn Invest. (2020) 32:664–74. doi: 10.1177/1040638720943584 32715975 PMC7488969

[43] Spring S Premathilake H DeSilva U Shili C Carter S Pezeshki A. Low protein-high carbohydrate diets alter energy balance, gut microbiota composition and blood metabolomics profile in young pigs. Sci Rep. (2020) 10:1–15. doi: 10.1038/s41598-020-60150-y 32094453 PMC7040010

[44] Benjamini Y Hochberg Y. Controlling the false discovery rate: a practical and powerful approach to multiple testing. J R Stat Soc Ser B. (1995) 57:289–300. doi: 10.1111/j.2517-6161.1995.tb02031.x

[45] Bilski J Mazur-ialy A Wojcik D Zahradnik-Bilska J Brzozowksi B Magierowski M. The role of intestinal alkaline phosphatase in inflammatory disorders of gastrointestinal tract. Mediat Inflamm. (2017) 2017:1–9. doi: 10.1155/2017/9074601 PMC5339520 28316376

[46] Hiney K Sypniewski L Rudra P Pezeshki A McFarlane D. Clinical health markers in dogs fed raw meat based or commercial extruded kibble diets. J Anim Sci. (2021) 99:skab133. doi: 10.1093/jas/skab133, PMID: 33939804 PMC8174467

[47] Castañeda S Ariza G Rincón-Riveros A Muñoz M Ramírez JD. Diet-induced changes in fecal microbiota composition and diversity in dogs (*Canis lupus familiaris*): A comparative study of BARF-type and commercial diets. Comp Immunol Microbiol Infect Dis. (2023) 98:102007. doi: 10.1016/j.cimid.2023.102007 37356167

[48] Schmidt M Unterer S Suchodolski JS Honneffer JB Guard B Lidbury JA. The fecal microbiome and metabolome differs between dogs fed bones and raw food (BARF) diets and dogs fed commercial diets. PLoS One. (2018) 13:e0201279. doi: 10.1371/journal.pone.0201279 30110340 PMC6093636

[49] Hooda S Boler BMV Kerr KR Dowd SE. The gut microbiome of kittens is affected by dietary protein: carbohydrate ratio and associated with blood metabolite and hormone concentrations. Br J Nutr. (2013) 109:1637–46. doi: 10.1017/S0007114512003479 22935193

[50] Bermingham EN Kittelmann S Henderson G Youngy W Roy NC Thomas DG. Five-week dietary exposure to dry diets alters the faecal bacterial populations in the domestic cat *(Felis catus)*. Br J Nutr. (2011) 106:S49–52. doi: 10.1017/S0007114511000572 22005435

[51] Sandri M Dal Monego S Conte G Sjorlin S Stefanon B. Raw meat based diet influences faecal microbiome and end products of fermentation in healthy dogs. BMC Vet Res. (2016) 13:1–11. doi: 10.1186/s12917-017-0981-z PMC5331737 28245817

[52] Janati AI Karp I Laprise C Sabri H Emami E. Detection of *Fusobacterium nucleatum* in feces and colorectal mucosa as a risk factor for colorectal cancer: a systematic review and meta-analysis. Syst Rev. (2020) 9:1–15. doi: 10.1186/s13643-020-01526-z 33272322 PMC7716586

[53] Butowski CF Moon CD Thomas DG Young W Bermingham EN. The effects of raw-meat diets on the gastrointestinal microbiota of the cat and dog: a review. N Z Vet J. (2022) 70:1–9. doi: 10.1080/00480169.2021.1975586 34463606

[54] Bermingham EN Young W Kittelmann S Kerr KR Swanson KS Roy NC. Dietary format alters fecal bacterial populations in the domestic cat *(Felis catus)*. Microbiology. (2013) 2:173–81. doi: 10.1002/mbo3.60 PMC3584222 23297252

[55] Middelbos IS Vester Boler BM Qu A White BA Swanson KS Fahey GC. Phylogenetic characterization of fecal microbial communities of dogs fed diets with or without supplemental dietary fiber using 454 pyrosequencing. PLoS One. (2010) 5:e9768. doi: 10.1371/journal.pone.0009768 20339542 PMC2842427

[56] Alexander C Cross TWL Devendran S Neumer F Theis S Ridlon JM. Effects of prebiotic inulin-type fructans on blood metabolite and hormone concentrations and faecal microbiota and metabolites in overweight dogs. Br J Nutr. (2018) 120:711–20. doi: 10.1017/S0007114518001952 30064535

[57] David LA Maurice CF Carmody RN Gootenberg DB Button JE Wolfe BE. Diet rapidly and reproducibly alters the human gut microbiome. Nature. (2014) 505:559–63. doi: 10.1038/nature12820 24336217 PMC3957428

[58] Wu GD Chen J Hoffmann C Bittinger K Chen YY Keilbaugh SA. Linking long-term dietary patterns with gut microbial enterotypes. Science. (2011) 334:105–8. doi: 10.1126/science.1208344 21885731 PMC3368382

[59] Klimenko NS Tyakht AV Popenko AS Vasiliev AS Altukhov IA Ischenko DS. Microbiome responses to an uncontrolled short-term diet intervention in the frame of the citizen science project. Nutrients. (2018) 10:E576. doi: 10.3390/nu10050576 PMC5986456 29738477

[60] Bermingham EN Kittelmann S Young W Kerr KR Swanson KS Roy NC. Post-weaning diet affects faecal microbial composition but not selected adipose gene expression in the cat (*Felis catus*). PLoS One. (2013) 8:e80992. doi: 10.1371/journal.pone.0080992 24312255 PMC3842929

[61] Kim J An J-U Kim W Lee S Cho S. Differences in the gut microbiota of dogs (*Canis lupus familiaris*) fed a natural diet or a commercial feed revealed by the Illumina MiSeq platform. Gut Pathog. (2017) 9:68. doi: 10.1186/s13099-017-0218-5 29201150 PMC5697093

[62] Kendig MD Hasebe K Tajaddini A Kaakoush NO Westbrook RF Morris MJ. The benefits of switching to a healthy diet on metabolic, cognitive, and gut microbiome parameters are preserved in adult rat offspring of mothers fed a high-fat. High Sugar Diet Mol Nutr Food Res. (2022) 67:2200318. doi: 10.1002/mnfr.20220031862 36271770 PMC10909468

[63] Harris EV de Roode JC Gerardo NM. Diet–microbiome–disease: investigating diet’s influence on infectious disease resistance through alteration of the gut microbiome. PLoS Pathog. (2019) 15:e1007891. doi: 10.1371/journal.ppat.1007891 31671152 PMC6822718

[64] Konturek PC Haziri D Brzozowski T Hess T Heyman S Kwiecien S. Emerging role of fecal microbiota therapy in the treatment of gastrointestinal and extragastrointestinal diseases. J Physiol Pharmacol. (2015) 66:486–91. 26348073

[65] Santos-Marcos JA Perez-Jimenez F Camargo A. The role of diet and intestinal microbiota in the development of metabolic syndrome. J Nutr Biochem. (2019) 70:1–27. doi: 10.1016/j.jnutbio.2019.03.017 31082615

[66] You I Kim MJ. Comparison of gut microbiota of 96 healthy dogs by individual traits: breed, age, and body condition score. Animals. (2021) 11:2432. doi: 10.3390/ani11082432 34438891 PMC8388711

[67] Reddy KE Kim HR Jeong JY So KM Lee S Ji SY. Impact of breed on the fecal microbiome of dogs under the same dietary condition. J Microbiol Biotechnol. (2019) 29:1947–56. doi: 10.4014/jmb.1906.06048 31601060

[68] Li Q Lauber C Czarnecki-Maulden G Pan Y Hannah SS. Effects of the dietary protein and carbohydrate ratio on gut microbiomes in dogs of different body conditions. MBio. (2017) 2017:10–1128. doi: 10.1128/mbio.01703-16 PMC5263242 28119466

[69] Puurunen J Ottka C Salonen M Niskanen JE Lohi H. Age, breed, sex and diet influence serum metabolite profiles of 2000 pet dogs. R Soc Open Sci. (2022) 9:211642. doi: 10.1098/rsos.211642 35223061 PMC8847897

[70] Kerr KR Vester Boler BM Morris CL Liu KJ Swanson KS. Apparent total tract energy and macronutrient digestibility and fecal fermentative end-product concentrations of domestic cats fed extruded, raw beef-based, and cooked beef-based diets. J Anim Sci. (2012) 90:515–22. doi: 10.2527/jas.2010-3266 22003235

[71] Neshovska H Shindarska Z. Comparative study of the digestibility of dry and raw food in dogs. Int J Vet Sci Anim Husb. (2021) 6:1–3.

[72] Law TH Volk HA Pan Y Zanghi B Want EJ. Metabolic perturbations associated with the consumption of a ketogenic medium-chain TAG diet in dogs with idiopathic epilepsy. Br J Nutr. (2018) 120:484–90. doi: 10.1017/S0007114518001617 30001753 PMC6137430

[73] Prasad K. Is there any evidence that AGE/sRAGE is a universal biomarker/risk marker for diseases? Mol Cell Biochem. (2019) 451:139–44. doi: 10.1007/s11010-018-3400-2 29961210

[74] Heilmann RM Allenspach K. Pattern-recognition receptors: signaling pathways and dysregulation in canine chronic enteropathies—brief review. J Vet Diag Invest. (2017) 29:781–7. doi: 10.1177/1040638717728545 28906208

[75] Bierhaus A Humpert PM Morcos M Wendt T Chavakis T Arnold B. Understanding RAGE, the receptor for advanced glycation end products. J Mol Med. (2005) 83:876–86. doi: 10.1007/s00109-005-0688-7 16133426

[76] Heilmann RM Otoni CC Jergens AE Grutzner N Suchodolksi JS Steiner JM. Systemic levels of the anti-inflammatory decoy receptor soluble RAGE (receptor for advanced glycation end products) are decreased in dogs with inflammatory bowel disease. Vet Immunol Immunopathol. (2014) 161:184–92. doi: 10.1016/j.vetimm.2014.08.003 25183017

[77] Liu W Hu D Huo H Zhang W Adiliaghdam F Morrison F. Intestinal alkaline phosphatase regulates tight junction protein levels. J Am Coll Surg. (2016) 222:1009–17. doi: 10.1016/j.jamcollsurg.2015.12.006 27106638 PMC5684582

[78] Goldberg R Austen W Zhang X Munene G Mostafa G Biswas S. Intestinal alkaline phosphatase is a gut mucosal defense factor maintained by enteral nutrition. Proc Nat Acad Sci. (2008) 105:3551–6. doi: 10.1073/pnas.0712140105 18292227 PMC2265168

[79] Lallès J-P. Intestinal alkaline phosphatase: novel functions and protective effects. Nutr Rev. (2014) 72:82–94. doi: 10.1111/nure.12082 24506153

[80] Liu Y Cavallaro P Kim B Liu T Wang H Kuhn G. A role for intestinal alkaline phosphatase in preventing liver fibrosis. Theranostics. (2021) 11:14. doi: 10.7150/thno.48468 33391458 PMC7681079

[81] Fujihashi K McGhee JR. Mucosal immunity and tolerance in the elderly. Mech Ageing Dev. (2004) 125:889–98. doi: 10.1016/j.mad.2004.05.009 15563935

[82] Schmucker DL Owen RL Outenreath R Thoreux K. Basis for the age-related decline in intestinal mucosal immunity. Clin Dev Immunol. (2003) 10:167–72. doi: 10.1080/10446670310001642168 14768948 PMC2485420

[83] Maria A Ayane L Putarouv T Loureiro B Neto B Casagrande M. The effect of age and carbohydrate and protein sources on digestibility, fecal microbiota, fermentation products, fecal IgA, and immunological blood parameters in dogs. J Anim Sci. (2017) 95:2452–66. doi: 10.2527/jas.2016.1302 28727033

[84] Cerutti A Rescigno M. The biology of intestinal immunoglobulin A responses. Immunity. (2008) 28:740–50. doi: 10.1016/j.immuni.2008.05.001 18549797 PMC3057455

[85] Macpherson A Khoo UY Forgacs I Philpott-Howard J Bjarnason I. Mucosal antibodies in inflammatory bowel disease are directed against intestinal bacteria. Gut. (1996) 38:365–75. doi: 10.1136/gut.38.3.365 8675088 PMC1383064

[86] Maeda S Ohno K Uchida K Nakashima K Fukushima K Tsukamoto A. Decreased immunoglobulin A concentrations in feces, duodenum, and peripheral blood mononuclear cells of dogs with inflammatory bowel disease. J Vet Intern Med. (2013) 27:47–55. doi: 10.1111/jvim.12023 23216572

[87] Lin R Chen H Shu W Sun M Fang L Shi Y. Clinical significance of soluble immunoglobulins A and G and their coated bacteria in feces of patients with inflammatory bowel disease. J Transl Med. (2018) 16:359. doi: 10.1186/s12967-018-1723-0 30558634 PMC6296095

