# Supplementary figures and images for: Fecal microbiota composition, serum metabolomics, and markers of inflammation in dogs fed a raw meat-based diet compared to those on a kibble diet
Source: Front Vet Sci. 2024 Apr 17;11:1328513. doi: 10.3389/fvets.2024.1328513 (PMC11061498; doi:10.3389/fvets.2024.1328513)

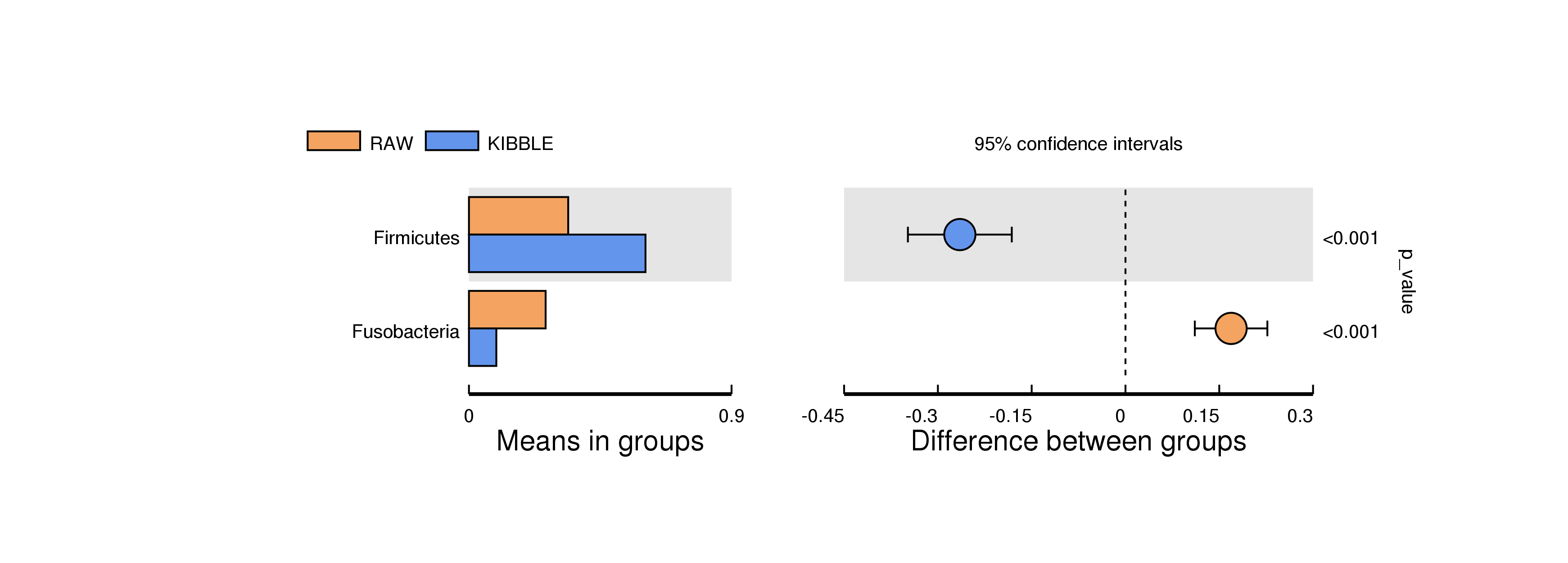

Supplement: SUPPLEMENTARY FIGURE S1 — T-test of between group variation in phylum. The left panel shows the species that differ significantly between groups. Each bar represents the mean value of the abundance in each group of the species showing significant difference between group. The right panel is the confidential interval of between group variation, with the bars indicating the 95% confidential interval. The center of the circle stands for the difference of the mean value. The color of the circle is in agree with the group whose mean value is higher. The p-value for the between group variation is on the right. [file Image_1.PNG]

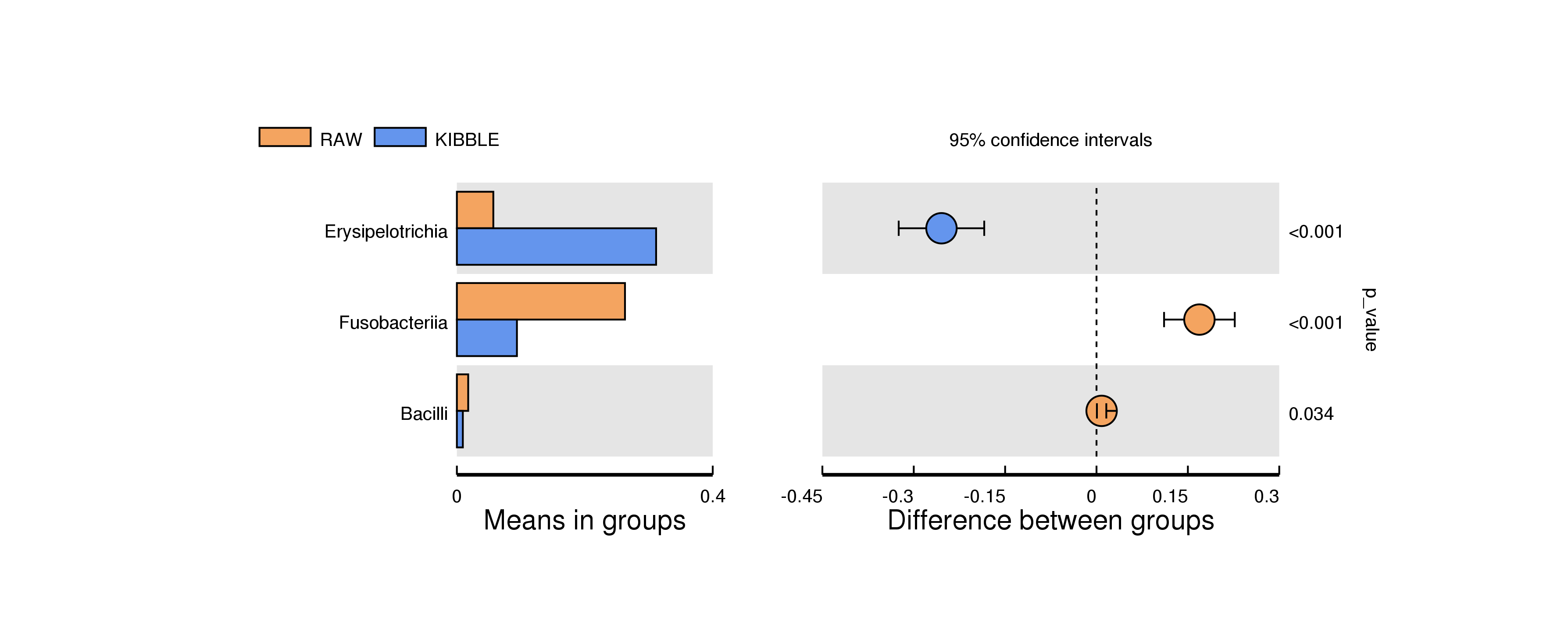

Supplement: SUPPLEMENTARY FIGURE S2 — T-test of between group variation in class. The left panel shows the species that differ significantly between groups. Each bar represents the mean value of the abundance in each group of the species showing significant difference between group. The right panel is the confidential interval of between group variation, with the bars indicating the 95% confidential interval. The center of the circle stands for the difference of the mean value. The color of the circle is in agree with the group whose mean value is higher. The p-value for the between group variation is on the right. [file Image_2.PNG]

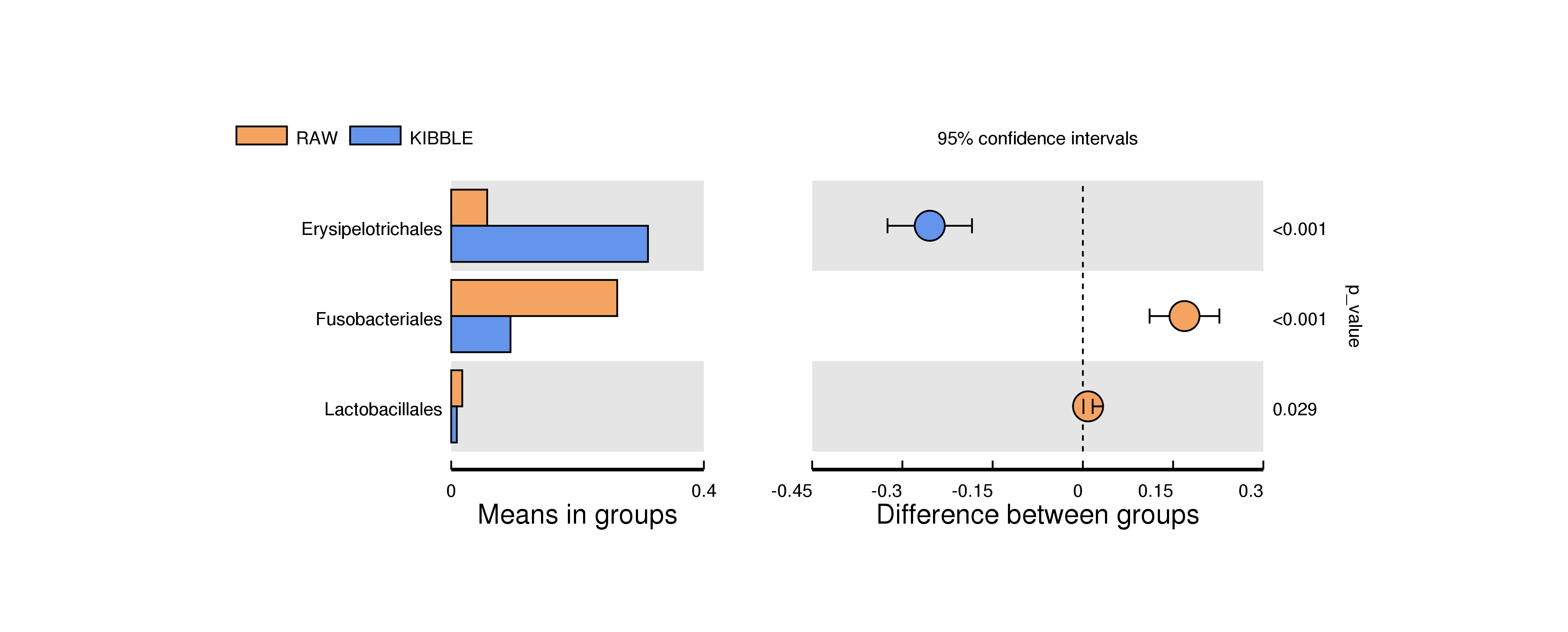

Supplement: SUPPLEMENTARY FIGURE S3 — T-test of between group variation in order. The left panel shows the species that differ significantly between groups. Each bar represents the mean value of the abundance in each group of the species showing significant difference between group. The right panel is the confidential interval of between group variation, with the bars indicating the 95% confidential interval. The center of the circle stands for the difference of the mean value. The color of the circle is in agree with the group whose mean value is higher. The p-value for the between group variation is on the right [file Image_3.PNG]

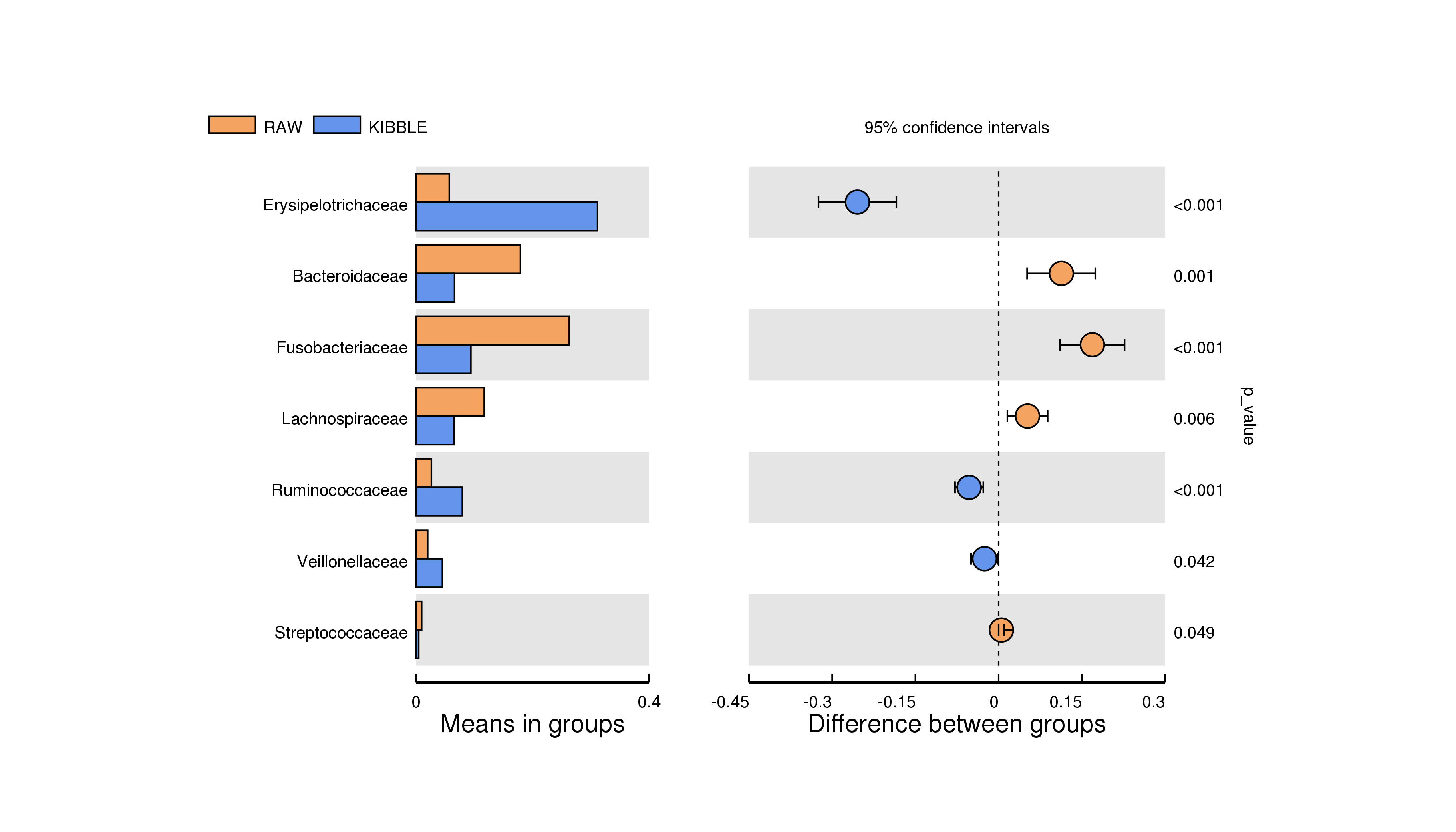

Supplement: SUPPLEMENTARY FIGURE S4 — T-test of between group variation in family. The left panel shows the species that differ significantly between groups. Each bar represents the mean value of the abundance in each group of the species showing significant difference between group. The right panel is the confidential interval of between group variation, with the bars indicating the 95% confidential interval. The center of the circle stands for the difference of the mean value. The color of the circle is in agree with the group whose mean value is higher. The p-value for the between group variation is on the right. [file Image_4.PNG]

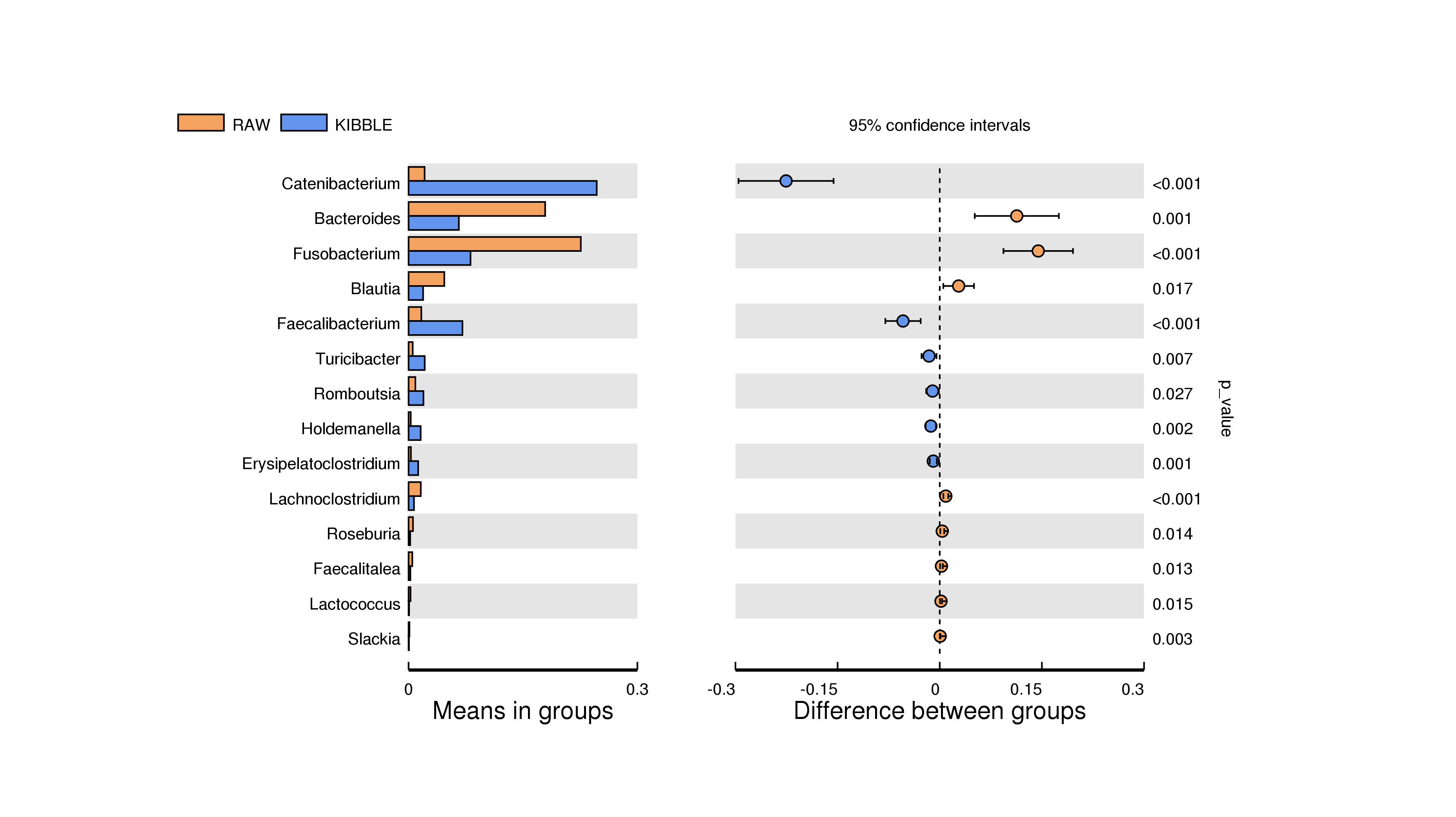

Supplement: SUPPLEMENTARY FIGURE S5 — T-test of between group variation in genus. The left panel shows the species that differ significantly between groups. Each bar represents the mean value of the abundance in each group of the species showing significant difference between group. The right panel is the confidential interval of between group variation, with the bars indicating the 95% confidential interval. The center of the circle stands for the difference of the mean value. The color of the circle is in agree with the group whose mean value is higher. The p-value for the between group variation is on the right. [file Image_5.PNG]

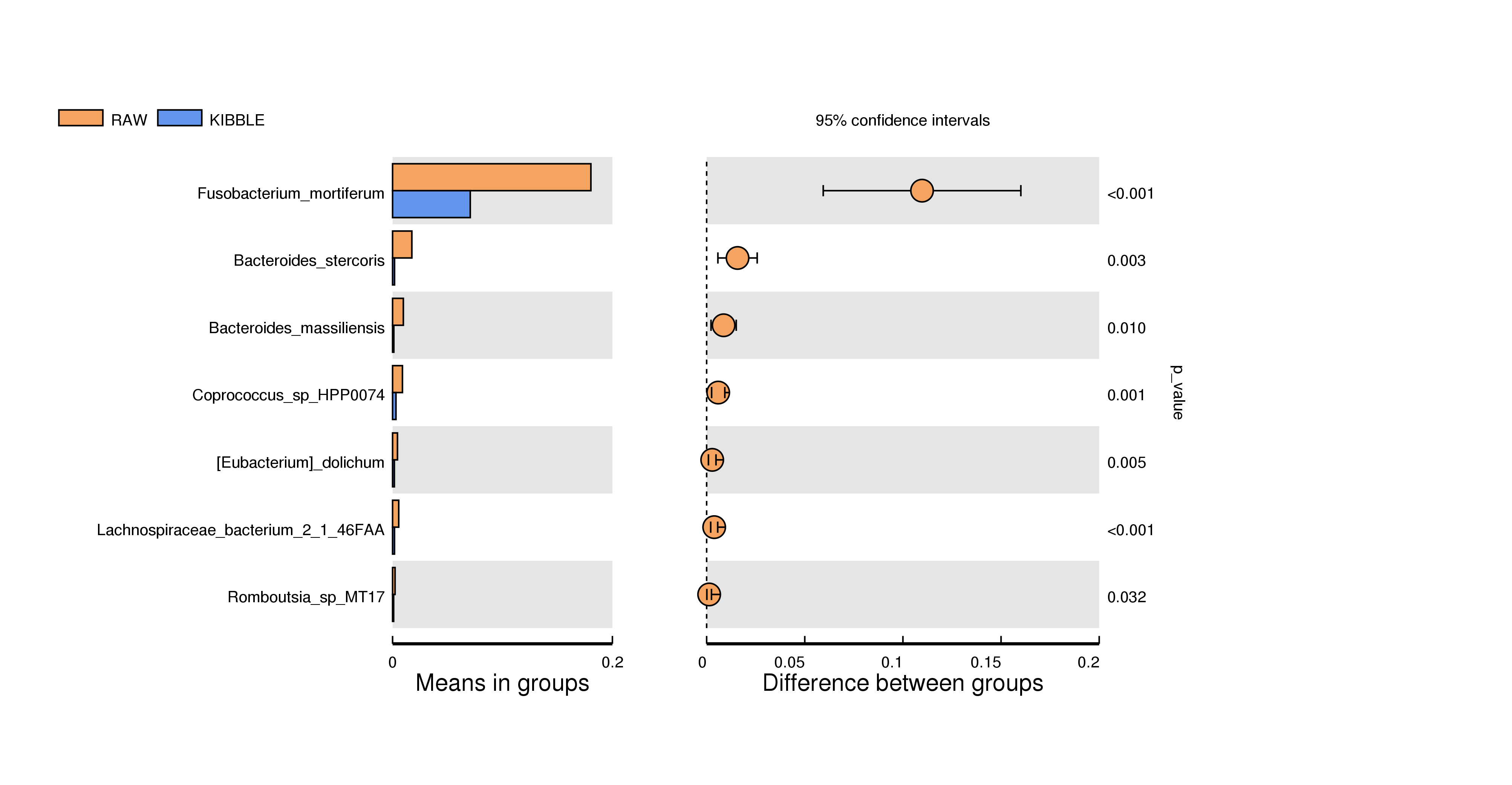

Supplement: SUPPLEMENTARY FIGURE S6 — T-test of between group variation in species. The left panel shows the species that differ significantly between groups. Each bar represents the mean value of the abundance in each group of the species showing significant difference between group. The right panel is the confidential interval of between group variation, with the bars indicating the 95% confidential interval. The center of the circle stands for the difference of the mean value. The color of the circle is in agree with the group whose mean value is higher. The p-value for the between group variation is on the right. [file Image_6.PNG]
