# Supplementary material for: Fecal microbiota composition, serum metabolomics, and markers of inflammation in dogs fed a raw meat-based diet compared to those on a kibble diet
Source: Front Vet Sci. 2024 Apr 17;11:1328513. doi: 10.3389/fvets.2024.1328513 (PMC11061498; doi:10.3389/fvets.2024.1328513)
Supplement: Supplementary file 7 [file Table_1.DOCX]

**Table S1. Analyzed nutrient composition of kibble or raw meat-based diets (RMBD) fed to client owned dogs on as fed and dry matter basis.**

|  | **KIBBLE ^1^** | | **RMBD ^2^** | |
| --- | --- | --- | --- | --- |
| Analyzed nutrient | As fed | Dry matter | As fed | Dry matter |
| ME (kcal/kg) | 3936 | 4296 | 1531 | 5280 |
| DM % | 91.61 |  | 29.0 |  |
| Crude protein, **%** | 28.2 | 30.78 | 14.40 | 49.66 |
| Fat (acid hydrolysis), **%** | 17.2 | 18.78 | 12.60 | 43.45 |
| Crude fiber, **%** | 1.96 | 2.14 | < 0.20 | <0.20 |
| Ash, **%** | 7.05 | 7.70 | 1.67 | 5.76 |
| Total sulfur, **%** | 0.36 | 0.39 | 0.14 | 0.48 |
| Total phosphorus, **%** | 1.04 | 1.14 | 0.34 | 1.17 |
| Total potassium, **%** | 0.73 | 0.80 | 0.20 | 0.69 |
| Total magnesium, **%** | 0.12 | 0.13 | 0.02 | 0.07 |
| Total calcium, **%** | 1.5 | 1.64 | 0.42 | 1.45 |
| Total sodium, **%** | 0.51 | 0.56 | 0.13 | 0.45 |
| Total iron, ppm^*^ | 297 | 324.20 | 29.80 | 102.76 |
| Total manganese, ppm^*^ | 81.7 | 89.18 | 4.40 | 15.17 |
| Total copper, ppm^*^ | 16.1 | 17.57 | 8.00 | 27.59 |
| Total zinc, ppm^*^ | 249 | 271.80 | 31.10 | 107.24 |
| Total starch, **%** | 29.63 | 32.34 | 0.11 | 0.38 |
| Lactose, % | ND | ND | ND | ND |
| Fructose, % | ND | ND | ND | ND |
| Glucose, % | ND | ND | ND | ND |
| Maltose, % | ND | ND | ND | ND |
| Sucrose, % | 1 | 1.09 | ND | ND |

^1^Kibble diet: 50;50 blend of Pro plan Shredded Beef with Rice and Shredded Chicken with rice:

Shredded Chicken with Rice: chicken, rice, whole grain wheat, poulty by product, soybean meal, beef fat, corn gluten meal, whole grain corn, egg product, fish meal, natural flavors, glycerin, wheat bran, calcium carbonate, mono and dicalcium phosphate, salt, soybean oil potassium chloride, fish oil, : zinc proteinate, manganese proteinate, ferrous sulfate, copper proteinate, calcium iodate, sodium selenite; niacin, vitamin A supplement, calcium pantothenate, thiamine mononitrate, vitamin B12 supplement, riboflavin supplement, pyridoxine hydrochloride, folic acid, vitamin D3 supplement, menadione sodium bisulfite, biotin, choline chloride, L-ascorbyl 2-polyphoshapte, dried Bacillus coagulans fermentation product, L-lysine monohydrochloride, garlic oil

Proplan beef: beef, rice, whole grain wheat, corn gluten meal, poultry by-product meal, soybean meal, whole grain corn, beef fat, egg product, dried beet pulp, natural flavors, glycerin, calcium carbonate, wheat bran, mono and dicalcium phosphate, soybean oil, salt, potassium chloride, Vitamins: vitamin E supplement, niacin, vitamin A supplement, calcium pantothenate, thiamine mononitrate, riboflavin supplement, pyridoxine hydrochloride, folic acid, vitamin d3 supplement, menadione sodium bisulfite, biotin, Minerals: zinc proteinate, manganese proteinate, ferrous sulfate, copper proteinate, calcium iodate, sodium selenite, choline chloride, L-ascorbyl 2-polyphoshapte, dried Bacillus coagulans fermentation product, garlic oil

^2^ RMBD: Poultry, beef, salmon by-product, egg product, beef tripe, beef liver, beef hears, beef kidneys, dicalcium phosphate, wheat germ oil, taurine, salt, potassium chloride, dried kelp, l-lysine, DL methionine, zinc sulfate, iron sulfate, magnesium oxide, yucca schidigera

Mineral analysis performed by ICAP using a wet digest procedure. *ppm, parts per millions;

ND: Below detection threshold
