# Supplementary material for: Fecal microbiota composition, serum metabolomics, and markers of inflammation in dogs fed a raw meat-based diet compared to those on a kibble diet
Source: Front Vet Sci. 2024 Apr 17;11:1328513. doi: 10.3389/fvets.2024.1328513 (PMC11061498; doi:10.3389/fvets.2024.1328513)
Supplement: Supplementary file 8 [file Table_2.DOCX]

**Table S2. Amino acids profile of kibble or raw meat-based diets (RMBD) (DM basis) and intake of amino acids (mg/kg bwt/d)**

|  | **Kibble % DM** | **Amino acid intake of kibble (mg/kg bwt)** | **RMBD** | **Amino acid intake of RMBD (mg/kg bwt)** |
| --- | --- | --- | --- | --- |
| Tryptophan | 0.29 | 31.3 | 0.59 | 42.4 |
| Cystine | 0.37 | 39.9 | 0.52 | 37.3 |
| Methionine | 0.53 | 57.1 | 1.38 | 99.1 |
| Alanine | 2.01 | 216.6 | 3.62 | 259.9 |
| Arginine | 1.71 | 184.3 | 3.55 | 254.9 |
| Aspartic acid | 2.41 | 259.7 | 4.69 | 336.7 |
| Glutamic acid | 5.15 | 555 | 7.21 | 517.7 |
| Glycine | 1.93 | 208.0 | 4.48 | 321.7 |
| Histidine | 0.67 | 72.2 | 1.31 | 94.1 |
| Isoleucine | 1.17 | 126.0 | 2.14 | 153.6 |
| Leucine | 2.83 | 305.0 | 4.00 | 287.2 |
| Phenylalanine | 1.42 | 153.0 | 2.17 | 155.8 |
| Proline | 2.15 | 231.7 | 3.07 | 220.4 |
| Serine | 1.39 | 149.8 | 2.28 | 163.7 |
| Threonine | 1.09 | 117.5 | 2.24 | 160.8 |
| Total Lysine | 1.39 | 163.8 | 4.07 | 292.2 |
| Tyrosine | 0.88 | 103.5 | 1.59 | 114.2 |
| Valine | 1.31 | 154.1 | 2.62 | 188.1 |
