# Supplementary material for: Fecal microbiota composition, serum metabolomics, and markers of inflammation in dogs fed a raw meat-based diet compared to those on a kibble diet
Source: Front Vet Sci. 2024 Apr 17;11:1328513. doi: 10.3389/fvets.2024.1328513 (PMC11061498; doi:10.3389/fvets.2024.1328513)
Supplement: Supplementary file 9 [file Table_3.DOCX]

**Table S3. Fatty acids profile of kibble or raw meat- based diets (RMBD) (DM basis) and intake of fatty acids (mg/kg bwt/d)**

|  | **Kibble DM** | **Kibble intake (mg/kg)** | **RMBD DM** | **RMBD intake**  **(mg/kg)** |
| --- | --- | --- | --- | --- |
| C12:0 (Lauric Acid) | 0.02 | 2.31 | 0.07 | 5.0 |
| C14:0 (Myristic acid) | 0.40 | 42.81 | 0.66 | 47.8 |
| C14:1 (Myristoleic acid) | 0.08 | 8.10 | 0.14 | 10.1 |
| C15:0 (Pentadecanoic acid) | 0.07 | 6.94 | 0.10 | 7.6 |
| C16:0 (Palmitic Acid) | 3.82 | 404.98 | 10.83 | 790.4 |
| C16:1 Omega 7 | 0.45 | 47.44 | 2.14 | 156.1 |
| C16:1 Total (Palmitoleic Acid + isomers) | 0.61 | 64.80 | 2.48 | 181.2 |
| C16:3 (Hexadecatrienoic Acid) | 0.09 | 9.26 | 0.14 | 10.1 |
| C17:0 (Margaric Acid) | 0.19 | 19.67 | 0.21 | 15.1 |
| C17:1 (Heptadecenoic Acid) | 0.10 | 10.41 | 0.10 | 7.6 |
| C18:0 (Stearic Acid) | 2.22 | 234.89 | 4.45 | 324.7 |
| C18:1 (Vaccenic acid) | 0.22 | 23.14 | 0.76 | 55.4 |
| C18:1 Omega 9 (Oleic Acid) | 5.32 | 563.50 | 15.55 | 1135.3 |
| C18:1, Total (Oleic Acid + isomers) | 6.30 | 667.63 | 16.76 | 1223.4 |
| C18:2 Omega 6 (Linoleic Acid) | 1.99 | 210.59 | 6.55 | 478.3 |
| C18:2, Total (Linoleic Acid + isomers) | 2.11 | 223.32 | 6.79 | 495.9 |
| C18:3 Omega 3 (Alpha Linolenic Acid) | 0.14 | 15.04 | 0.41 | 30.2 |
| C18:3, Total (Linolenic Acid + isomers) | 0.15 | 16.20 | 0.45 | 32.7 |
| C18:4 Total (Octadecatetraenoic Acid) | ND | ND | ND | ND |
| C20:0 (Arachidic Acid) | 0.03 | 3.47 | ND | ND |
| C20:1 Omega 9 (Gondoic Acid) | 0.04 | 4.63 | 0.17 | 12.6 |
| C20:1 Total (Gondoic Acid + isomers) | 0.07 | 6.94 | 0.28 | 20.1 |
| C20:2 Omega 6 | ND | ND | 0.07 | 5.0 |
| C20:2 Total (Eicosadienoic Acid) | ND | ND | 0.07 | 5.0 |
| C20:3 Omega 6 | ND | ND | 0.10 | 7.6 |
| C20:3, Total (Eicosatrienoic Acid) | ND | ND | 0.14 | 10.1 |
| C20:4 Omega 6 (Arachidonic Acid) | 0.04 | 4.63 | 0.34 | 25.2 |
| C20:4, Total (Eicosatetraenoic Acid) | 0.04 | 4.63 | 0.34 | 25.2 |
| C20:5 Omega 3 (Eicosapentaenoic Acid) | 0.03 | 3.47 | 0.14 | 10.1 |
| C22:1 Omega 9 (Erucic Acid) | ND | ND | 0.14 | 10.1 |
| C22:1 Total (Erucic Acid + isomers) | ND | ND | 0.17 | 12.6 |
| C22:4 Docosatetraenoic Omega 6 | ND | ND | 0.07 | 5.0 |
| C22:5 Docosapentaenoic Omega 3 | ND | ND | 0.10 | 7.6 |
| C22:5 Total (Docosapentaenoic Acid) | ND | ND | 0.10 | 7.6 |
| C22:6 Docosahexaenoic Omega 3 | 0.03 | 3.47 | 0.21 | 15.1 |
| Total Omega 3 Isomers | 0.24 | 25.46 | 0.90 | 65.4 |
| Total Omega 5 Isomers | 0.08 | 8.10 | ND | ND |
| Total Omega 6 Isomers | 2.07 | 219.84 | 7.21 | 526.1 |
| Total Omega 7 Isomers | 0.68 | 71.74 | 2.93 | 214.0 |
| Total Omega 9 Isomers | 5.38 | 570.44 | 15.93 | 1163.0 |
| Total Monounsaturated Fatty Acids | 6.45 | 683.83 | 19.55 | 1427.3 |
| Total Polyunsaturated Fatty Acids | 2.47 | 261.50 | 8.41 | 614.2 |
| Total Saturated Fatty Acids | 6.78 | 718.55 | 16.45 | 1200.7 |
| Total Trans Fatty Acids | 0.78 | 82.15 | 0.52 | 37.8 |
| Total Fat as Triglycerides | 17.25 | 1828.18 | 47.00 | 3431.0 |
| Total Fatty Acids | 16.48 | 1747.19 | 44.90 | 3277.4 |
