# Supplementary material for: Fecal microbiota composition, serum metabolomics, and markers of inflammation in dogs fed a raw meat-based diet compared to those on a kibble diet
Source: Front Vet Sci. 2024 Apr 17;11:1328513. doi: 10.3389/fvets.2024.1328513 (PMC11061498; doi:10.3389/fvets.2024.1328513)
Supplement: Supplementary file 10 [file Table_4.DOCX]

**Table S4. Mean ±SD nutrient intake of kibble or raw meat-based diets (RMBD) per kg of bwt.**

|  | **Kibble** | **RMBD** |
| --- | --- | --- |
| ME kcal | 46.3 ± 10.6 | 37.9 ± 8.3 |
| Crude Protein (g)/ | 3.3 ± 0.75 | 3.6 ±0.79 |
| Fat (acid hydrolysis) (g) | 2.0 ± 0.46 | 3.2 ± 0.69 |
| Crude Fiber (g) | 0.2 ± 0.05 | ND |
| Total starch (g) | 3.4 ± 0.79 | 0.03 ± 0.01 |
| Calcium (mg) | 173.5 ± 40.1 | 105.6 ± 23.0 |
| Phosphorous (mg) | 120.6 ± 27.8 | 85.2 ± 18.6 |
| Magnesium mg | 13.8 ± 3.18 | 5.1 ± 1.1 |
| Copper mg | 0.19 ± 0.04 | 0.20 ± 0.04 |
| Zinc mg | 2.9 ± 0.66 | 0.78 ± 0.17 |

ND: Below detection threshold
