# Supplementary material for: Fecal microbiota composition, serum metabolomics, and markers of inflammation in dogs fed a raw meat-based diet compared to those on a kibble diet
Source: Front Vet Sci. 2024 Apr 17;11:1328513. doi: 10.3389/fvets.2024.1328513 (PMC11061498; doi:10.3389/fvets.2024.1328513)
Supplement: Supplementary file 17 [file Table_11.docx]

| **Table S11**. Non-significant, identified serum metabolomic profile in dogs fed with kibble or raw meat-based diets (RMBD.) Results are listed as mean ± SD. If data did not pass normality testing (Shapiro Wilks), results listed as median (IQR). Serum metabolite concentration was compared by multiple linear regression, with diet group, age and BCS as independent variables. P values were corrected for multiple comparisons using Benjamini-Hochberg procedure to control False Discovery Rate. | | | |
| --- | --- | --- | --- |
| **Metabolites** | **Diets** | | **Corrected P-Value** |
|  | **Kibble** | **RMBD** |  |
| 1,3-dihydroxypyridine | 202 ± 41 | 192 ± 39 | 0.650 |
| 1-monopalmitin | 730 ± 172 | 658 ± 209 | 0.134 |
| 1-monostearin | 2453 (2080-2886) | 2139 (1745-2829) | 0.110 |
| 2-deoxytetronic acid | 488 ± 124 | 516 ± 121 | 0.649 |
| 2-picolinic acid | 3278 (1638-5800) | 3615 (1760-5462) | 0.413 |
| 2,3-dihydroxybutanoic acid | 666 (510-857) | 661 (480-862) | 0.694 |
| 2,5-dihydroxypyrazine | 1561 (1312-1834) | 1389 (1218-1880) | 0.613 |
| 3-phosphoglycerate | 647 (515-866) | 589 (425-921) | 0.417 |
| 5-aminovaleric acid | 1222 (807-1658) | 1718 (83-2769) | 0.292 |
| 6-deoxyglucose | 2143 (1955-2408) | 2029 (1829-2352) | 0.185 |
| Aconitic acid | 872 ± 269 | 809 ± 221 | 0.202 |
| Adenosine-5-monophosphate | 369 (258-621) | 370 (236-552) | 0.204 |
| Alanine | 362555 ± 120103 | 315503 ± 71628 | 0.708 |
| Allantoic acid | 5589 (4478-6949) | 5567 (3812-7883) | 0.634 |
| Alloxanoic acid | 369 (292-724) | 434 (241-598) | 0.959 |
| Alpha-ketoglutarate | 6816 ± 1527 | 6981 ± 2058 | 0.977 |
| Anthranilic acid | 302 (223-408) | 379 (332-514) | 0.329 |
| Asparagine | 6669 (5113-7776) | 5261 (4176-7487) | 0.197 |
| Azelaic acid | 331 ± 116 | 285 ± 103 | 0.100 |
| Benzoic acid | 7533 (6443-8541) | 7558 (6820-9332) | 0.700 |
| Cholesterol | 683641 ± 92316 | 650219 ± 120104 | 0.498 |
| Citric acid | 256538 ± 52441 | 260338 ± 62029 | 0.796 |
| Citrulline | 9918 (8309-11005) | 9875 (8074-11464) | 0.692 |
| Conduritol-beta-epoxide | 3073 ± 1223 | 3278 ± 930 | 0.603 |
| Creatine | 55815 ± 17092 | 66566 ± 15214 | 0.459 |
| Cysteine | 1177 (981-1392) | 1219 (1091-1404) | 0.643 |
| Fucose | 362 ± 105 | 334 ± 114 | 0.412 |
| Glucose-1-phosphate | 3082 ± 789 | 2517 ± 865 | 0.400 |
| Glucuronic acid | 3123 (2283-4163) | 2983 (1983-4787) | 0.60 |
| Glyceric acid | 3856 ± 738 | 3597 ± 766 | 0.162 |
| Glycerol-3-galactoside | 338 (257-475) | 304 (263-462) | 0.977 |
| Glycerol-alpha-phosphate | 1903 ± 326 | 1907 ± 312 | 0.977 |
| Glycine | 192882 ± 33718 | 189714 ± 40401 | 0.685 |
| Glycolic acid | 7252 (6231-8625) | 6508 (6039-8843) | 0.757 |
| Histidine | 4833 (3371-12522) | 4733 (3837-7487) | 0.997 |
| Hydroquinone | 3688 (2439-8073) | 5440 (3700-9762) | 0.796 |
| Hypoxanthine | 1138 (602-1839) | 498 (300-882) | 0.158 |
| Indole-3-acetate | 1408 (1078-1849) | 1003 (676-1440) | 0.304 |
| Inosine | 335 (129-737) | 169 (68-258) | 0.163 |
| Isolinoleic acid | 316 (263-336) | 242 (204-275) | 0.163 |
| Lactic acid | 574305 ± 248759 | 461510 ± 206292 | 0.226 |
| Lactulose | 267 (215-296) | 265 (214-350) | 0.426 |
| Lauric acid | 7832 (6063-9836) | 7964 (6567-9674) | 0.877 |
| Malic acid | 2221 (1936-2404) | 1889 (1548-2497) | 0.215 |
| Mannitol | 3947 (3465-4659) | 4167 (3548-4949) | 0.643 |
| Methanolphosphate | 7306 (6317-8202) | 6705 (5217-8031) | 0.142 |
| Methionine | 56090 ± 12132 | 62163 ± 12959 | 0.623 |
| Methionine sulfoxide | 10730 ± 2489 | 12244 ± 2826 | 0.202 |
| N-acetylmannosamine | 303 ± 95 | 236 ± 104 | 0.127 |
| Nicotinic acid | 9767 (5893-169550 | 15636 ± 8820-24544) | 0.314 |
| Orotic acid (B13) | 291 ± 109 | 306 ± 111 | 0.633 |
| Oxalic acid | 55268 (36573-128887) | 94775 (57648-141267) | 0.431 |
| Palmitic acid | 105963 ± 26128 | 124061 ± 41328 | 0.341 |
| Palmitoleic acid | 2747 (1295-4087) | 3542 (2480-5742) | 0.329 |
| Parabanic acid | 1550 (814-3937) | 2701 (1538-4399) | 0.358 |
| Phenol | 10511 (8911-12158) | 11149 (9380-13385) | 0.532 |
| Phenylethylamine | 8130 (6527-10152) | 8713 (7442-13670) | 0.748 |
| Phosphate | 433133 ± 99532 | 421752 ± 81525 | 0.643 |
| Phosphoenolpyruvate | 1597 (851-2102) | 1069 (598-1672) | 0.197 |
| Proline | 152340 ± 58458 | 127792 ± 56406 | 0.102 |
| Pyrophosphate | 5924 (5005-6509) | 5899 (5029-6504) | 0.820 |
| Pyruvic acid | 22919 (12944-41456) | 26099 (14772-35472) | 0.976 |
| Serine | 189906 ± 44714 | 236232 ± 65689 | 0.102 |
| Succinic acid | 2496 (2056-3295) | 2337 (1706-2884) | 0.455 |
| Threonine | 62592 (55502-90851) | 83106 (62951-99820) | 0.202 |
| Thymidine | 5053 (4129-6958) | 5501 (4613-6680) | 0.393 |
| Tyrosol | 268 (209-360) | 397 (265-693) | 0.115 |
| Uracil | 393 (342-487) | 343 (298-463) | 0.757 |
| Uric acid | 1832 (1348-2097) | 1592 (1417-1924) | 0.757 |
| Xylitol | 1201 ± 319 | 1250 ± 297 | 0.484 |
| Xylose | 3166 (2940-4083) | 3553 (2909-3829) | 0.505 |
| Xylulose | 610 (508-678) | 523 (492-616) | 0.115 |
| Zymosterol | 806 ± 335 | 689 ± 276 | 0. 700 |
|  | | | |
