# Supplementary material for: Fecal microbiota composition, serum metabolomics, and markers of inflammation in dogs fed a raw meat-based diet compared to those on a kibble diet
Source: Front Vet Sci. 2024 Apr 17;11:1328513. doi: 10.3389/fvets.2024.1328513 (PMC11061498; doi:10.3389/fvets.2024.1328513)
Supplement: Supplementary file 18 [file Table_12.DOCX]

8 Methods

Novogene Co., Ltd

8.1 Sequencing preparation

**1 Genomic DNA extraction**

Total genome DNA from samples was extracted using CTAB/SDS method. DNA concentration and purity was monitored on 1% agarose gels. According to the concentration, DNA was diluted to 1ng/μL using sterile water.

**2 Amplicon Generation**

16S rRNA/18SrRNA/ITS genes of distinct regions (16SV4/16SV3/16SV3-V4/16SV4-V5, 18S V4/18S V9, ITS1/ITS2, Arc V4) were amplified used specific primer (e.g. 16S V4: 515F-806R, 18S V4: 528F-706R, 18S V9: 1380F-1510R, et. al ) with the barcode. All PCR reactions were carried out with Phusion® High-Fidelity PCR Master Mix (New England Biolabs).

**3 PCR Products quantification and qualification**

Mix same volume of 1X loading buffer (contained SYB green) with PCR products and operate electrophoresis on 2% agarose gel for detection. Samples with bright main strip between 400bp-450bp were chosen for further experiments.

**4 PCR Products Mixing and Purification**

PCR products was mixed at equal density ratios. The mixed PCR products were purified with Qiagen Gel Extraction Kit (Qiagen, Germany).

The libraries generated with NEBNext® UltraTM DNA Library Prep Kit for Illumina and quantified via Qubit and Q-PCR, would be analysed by Illumina platform.

8.2 Information analysis

**1 Sequencing data processing**

Paired-end reads was assigned to samples based on their unique barcodes and truncated by cutting off the barcode and primer sequences. Paired-end reads were merged using FLASH (V1.2.7)[20] (see details <http://ccb.jhu.edu/software/FLASH/>), a very fast and accurate analysis tool, which was designed to merge paired-end reads when at least some of the reads overlap the read generated from the opposite end of the same DNA fragment, and the splicing sequences were called raw tags. Quality filtering on the raw tags were performed under specific filtering conditions to obtain the high-quality clean tags[21] according to the Qiime (V1.7.0)[22] (see details <http://qiime.org/scripts/split_libraries_fastq.html>) quality controlled process.

The tags were compared with the reference database (Gold database, see details <http://drive5.com/uchime/uchime_download.html>) using UCHIME algorithm (UCHIME Algorithm, see details <http://www.drive5.com/usearch/manual/uchime_algo.html>)[23] to detect chimera sequences (see details <https://drive5.com/usearch/manual/chimeras.html>). And then the chimera sequences were removed[24]. Then the Effective Tags finally obtained.

**2 OTU cluster and Taxonomic annotation**

Sequences analysis were performed by Uparse software (Uparse v7.0.1001, see details <http://drive5.com/uparse/>)[25] using all the effective tags. Sequences with ≥97% similarity were assigned to the same OTUs. Representative sequence for each OTU was screened for further annotation.

For each representative sequence, Mothur software was performed against the SSUrRNA database of SILVA Database (see details <http://www.arb-silva.de/>)[26]for species annotation at each taxonomic rank (Threshold:0.8~1)[27] (kingdom, phylum, class, order, family, genus, species).

To obtain the phylogenetic relationship of all OTUs representative sequences, the MUSCLE[28] (Version 3.8.31, see details <http://www.drive5.com/muscle/>) can compare multiple sequences rapidly.

OTUs abundance information were normalized using a standard of sequence number corresponding to the sample with the least sequences. Subsequent analysis of alpha diversity and beta diversity were all performed basing on this output normalized data.

**3 Alpha Diversity**

Alpha diversity is applied in analyzing complexity of biodiversity for a sample through 6 indices, including Observed-species, Chao1, Shannon, Simpson, ACE, Good-coverage. All these indices in our samples were calculated with QIIME (Version 1.7.0) and displayed with R software (Version 2.15.3).

Alpha Diversity Indices:

Community richness indices:

Chao - the Chao1 estimator (see details <http://scikit-bio.org/docs/latest/generated/skbio.diversity.alpha.chao1.html#skbio.diversity.alpha.chao1>);

ACE - the ACE estimator (see details <http://scikit-bio.org/docs/latest/generated/skbio.diversity.alpha.ace.html#skbio.diversity.alpha.ace>);

Community diversity indices:

Shannon - the Shannon index (see details <http://scikit-bio.org/docs/latest/generated/skbio.diversity.alpha.shannon.html#skbio.diversity.alpha.shannon>);"

Simpson - the Simpson index (see details <http://scikit-bio.org/docs/latest/generated/skbio.diversity.alpha.simpson.html#skbio.diversity.alpha.simpson>);

The index of sequencing depth:

Coverage - the Good’s coverage (see details <http://scikit-bio.org/docs/latest/generated/skbio.diversity.alpha.goods_coverage.html#skbio.diversity.alpha.goods_coverage>);

The index of phylogenetic diversity:

PD_whole_tree - PD_whole_tree index (see details <http://scikit-bio.org/docs/latest/generated/skbio.diversity.alpha.faith_pd.html?highlight=pd#skbio.diversity.alpha.faith_pd>)

**4 Beta Diversity**

Beta diversity analysis was used to evaluate differences of samples in species complexity, Beta diversity on both weighted and unweighted unifrac were calculated by QIIME software (Version 1.7.0). Cluster analysis was preceded by principal component analysis (PCA), which was applied to reduce the dimension of the original variables using the FactoMineR package and ggplot2 package in R software (Version 2.15.3). Principal Coordinate Analysis (PCoA) was performed to get principal coordinates and visualize from complex, multidimensional data. A distance matrix of weighted or unweighted unifrac among samples obtained before was transformed to a new set of orthogonal axes, by which the maximum variation factor is demonstrated by first principal coordinate, and the second maximum one by the second principal coordinate, and so on. PCoA analysis was displayed by WGCNA package, stat packages and ggplot2 package in R software (Version 2.15.3). Unweighted Pair-group Method with Arithmetic Means (UPGMA) Clustering was performed as a type of hierarchical clustering method to interpret the distance matrix using average linkage and was conducted by QIIME software (Version 1.7.0).

LEfSe analysis was conducted by LEfSe software. Metastat was calculated by R software. P-value was calculated by method of permutation test while q-value was calculated by method of Benjamini and Hochberg False Discovery Rate[29]. Anosim, MRPP and Adonis were performed by R software (Vegan package: anosim function, mrpp function and adonis function). AMOVA was calculated by mothur using amova function. T_test and drawing were conducted by R software.

9 References

[1] Caporaso, J. Gregory, et al. Global patterns of 16S rRNA diversity at a depth of millions of sequences per sample. Proceedings of the National Academy of Sciences 108.Supplement 1 (2011): 4516-4522.

[2] Youssef, Noha, et al. Comparison of species richness estimates obtained using nearly complete fragments and simulated pyrosequencing-generated fragments in 16S rRNA gene-based environmental surveys. Applied and environmental microbiology 75.16 (2009): 5227-5236.

[3] Hess, Matthias, et al. Metagenomic discovery of biomass-degrading genes and genomes from cow rumen. Science 331.6016 (2011): 463-467.

[4] Asnicar F, Weingart G, Tickle T L, et al. Compact graphical representation of phylogenetic data and metadata with GraPhlAn[J]. PeerJ, 2015.

[5] DeSantis, T. Z., et al. NAST: a multiple sequence alignment server for comparative analysis of 16S rRNA genes. Nucleic acids research 34.suppl 2 (2006): W394-W399.

[6] Ondov, Brian D., Nicholas H. Bergman, and Adam M. Phillippy. Interactive metagenomic visualization in a Web browser. BMC bioinformatics 12.1 (2011): 385.

[7] Li, Bing, et al. Characterization of tetracycline resistant bacterial community in saline activated sludge using batch stress incubation with high-throughput sequencing analysis. Water research 47.13 (2013): 4207-4216.

[8] Lundberg, Derek S., et al. Practical innovations for high-throughput amplicon sequencing.Nature methods 10.10 (2013): 999-1002.

[9] Lozupone, Catherine, and Rob Knight. UniFrac: a new phylogenetic method for comparing microbial communities. Applied and environmental microbiology 71.12 (2005): 8228-8235.

[10] Lozupone, Catherine, et al. UniFrac: an effective distance metric for microbial community comparison. The ISME journal 5.2 (2011): 169.

[11] Lozupone, Catherine A., et al. Quantitative and qualitative β diversity measures lead to different insights into factors that structure microbial communities. Applied and environmental microbiology 73.5 (2007): 1576-1585.

[12] Avershina, Ekaterina, Trine Frisli, and Knut Rudi. De novo Semi-alignment of 16S rRNA Gene Sequences for Deep Phylogenetic Characterization of Next Generation Sequencing Data. Microbes and Environments 28.2 (2013): 211-216.

[13] Magali Noval Rivas, PhD, Oliver T. Burton, et al. A microbita signature associated with experimental food allergy promotes allergic senitization and anaphylaxis. The Journal of Allergy and Clinical Immunology.Volume 131, Issue 1, Pages 201-212, January 2013.

[14] Anderson, M.J. 2001. A new method for non-parametric multivariate analysis of variance. Austral Ecology, 26: 32-46.

[15] McArdle, B.H. and M.J. Anderson. 2001. Fitting multivariate models to community data: A comment on distance-based redundancy analysis. Ecology, 82: 290-297.

[16] Warton, D.I., Wright, T.W., Wang, Y. 2012. Distance-based multivariate analyses confound location and dispersion effects. Methods in Ecology and Evolution, 3, 89-101.

[17] Zapala, M.A. and N.J. Schork. 2006. Multivariate regression analysis of distance matrices for testing associations between gene expression patterns and related variables. Proceedings of the National Academy of Sciences, USA, 103:19430-19435.

[18] Excoffier, L., Smouse, P.E. and Quattro, J.M. (1992) Analysis of molecular variance inferred from metric distances among DNA haplotypes: application to human mitochondrial DNA restriction data. Genetics, 131, 479-491.

[19] Magoč, Tanja, and Steven L. Salzberg. FLASH: fast length adjustment of short reads to improve genome assemblies. Bioinformatics 27.21 (2011): 2957-2963.

[20] Bokulich, Nicholas A., et al. Quality-filtering vastly improves diversity estimates from Illumina amplicon sequencing. Nature methods 10.1 (2013): 57-59.

[21] Caporaso, J. Gregory, et al. QIIME allows analysis of high-throughput community sequencing data. Nature methods 7.5 (2010): 335-336.

[22] Edgar, Robert C., et al. UCHIME improves sensitivity and speed of chimera detection. Bioinformatics 27.16 (2011): 2194-2200.

[23] Haas, Brian J., et al. Chimeric 16S rRNA sequence formation and detection in Sanger and 454-pyrosequenced PCR amplicons. Genome research 21.3 (2011): 494-504.

[24] Edgar, Robert C. UPARSE: highly accurate OTU sequences from microbial amplicon reads. Nature methods 10.10 (2013): 996-998.

[25] Wang, Qiong, et al. Naive Bayesian classifier for rapid assignment of rRNA sequences into the new bacterial taxonomy. Applied and environmental microbiology 73.16 (2007): 5261-5267.

[26] Quast C, Pruesse E, et al.The SILVA ribosomal RNA gene database project: improved data processing and web-based tools. Nucl. Acids Res. (2013) : D590-D596.

[27] MUSCLE: multiple sequence alignment with high accuracy and high throughputEdgar, 2004

[28] White, James Robert, Niranjan Nagarajan, and Mihai Pop. Statistical methods for detecting differentially abundant features in clinical metagenomic samples. PLoS computational biology 5.4 (2009): e1000352.
